# Basic adsorption heat exchanger theory for performance prediction of adsorption heat pumps

**DOI:** 10.1016/j.isci.2023.108432

**Published:** 2023-11-11

**Authors:** Andreas Velte-Schäfer, Eric Laurenz, Gerrit Füldner

**Affiliations:** 1 Fraunhofer Institute for Solar Energy Systems (ISE), Heidenhofstrasse 2, 79110 Freiburg, Germany

**Keywords:** Applied sciences, Energy Modelling, Heat Transfer

## Abstract

Adsorption modules are the core components of thermally driven adsorption heat pumps and chillers. Due to the transient nature of the adsorption and desorption processes, usually complicated numerical models are used for prediction of efficiency and heat flow rates. In this research article, we suggest a radically simplified calculation based on splitting up the ad- and desorption half cycle into a transient, strongly non-isothermal switching phase and a quasi-isothermal phase. In the quasi-isothermal phase, the heat flow rates can be calculated with relationship between temperature effectiveness (ϵ) and number of transfer units (NTU). Effective thermal resistances account for the heat and mass transfer processes. The prediction quality of our simple calculation in terms of heat flow rates is within ±20% compared with experimental data of two different sorption modules. The suggested method and its experimental validation lay the foundation of a basic adsorption heat exchanger theory.

## Highlights

• Performance prediction of adsorption heat pumps: radically simplified calculation
• Temperature effectiveness (ϵ) and number of transfer units (NTU) approach is used
• Prediction quality of heat flow rates is ±20% compared with experimental results
• Calculation based on algebraic equations, which are suited for microcontrollers

## Graphical abstract

**Figure:**
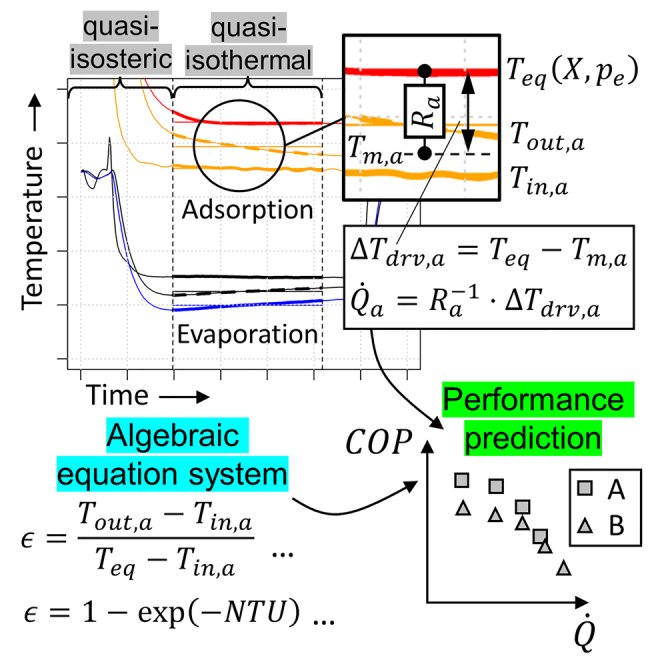

## Teaser

Applied sciences; Energy Modeling; Heat Transfer

## Introduction

Thermally driven heat transformation can contribute to reducing CO_2_ emissions and the demand for fossil fuels by utilizing waste heat as driving heat source or abundant environmental energy to supply the evaporator.^1^ Possible applications include waste-heat-driven cooling,^2^^,^^3^^,^^4^ gas-fired adsorption heat pumps,^5^^,^^6^^,^^7^ data center cooling,^8^ generating heat from cold,^9^^,^^10^ heat transformation to upgrade waste heat,^11^ thermal management of electronic devices,^12^ and desalination.^13^

Adsorptive heat transformation devices usually consist of an adsorption heat exchanger (ADHX), evaporator (E), and condenser (C).^14^ Evaporator and condenser might be one combined heat exchanger (EC) that switches its function during the cycle.^15^ The assembly of these components (ADHX, E, and C or ADHX and EC) is called an adsorption module.

The heat exchanger design procedure involves the use of complicated numerical models accounting for the coupled heat and mass transfer as recent examples of Khatibi et al.^16^ and Gibelhaus et al.^17^ show. With such a numerical model the heat flow rates are calculated from transient temperature curves. The transient heat flow rates are then averaged over the half cycle times and integrated, yielding the amounts of heat to calculate the thermal efficiency. The mean heat flow rates and thermal efficiency serve as key performance indicators for the design evaluation.^18^ The procedure of setting up a transient numerical model and post-processing the transient data to calculate the key performance indicators is time-consuming and computationally expensive.

To reduce the complexity and to discuss their results, Zheng et al.,^19^ later Alam et al.,^20^^,^^21^ and Miyazaki et al.^22^ evaluate dimensionless numbers like Biot-number or number of transfer units (NTU) that are related to the heat transfer processes within ADHX and EC. These works show that basic thermodynamic knowledge on heat exchangers as presented by Shah and Sekuliḉ^23^ might be helpful in developing a much simpler calculation of key performance indicators for adsorptive heat transformation devices. This basic heat exchanger theory allows for a prediction of heat flow rates with given inlet temperatures and capacity flowrates. These predictions are based on temperature effectiveness ϵ, the number of transfer units NTU, and heat exchanger specific ϵ−NTU relationships. However, applying basic heat exchanger theory is limited to steady state processes with constant temperatures. Since adsorption and desorption are transient processes and the temperature of the heat exchangers might switch between 30°C and 90°C in a cycle, basic heat exchanger theory and adsorptive heat transformation do not appear to be a good match. This obstacle can be overcome by splitting up the adsorption and desorption half cycles into a switching phase and quasi-isothermal phases. Then, the quasi-isothermal phase can be treated with basic heat exchanger theory with all its advantages. But with given inlet temperatures and capacity flow rates there is still one part missing: the overall heat conductance UA. In case of liquid-liquid heat exchangers, the overall heat conductance UA usually can be calculated with Nusselt-Reynolds correlations that only depend on flow rates, temperatures, and the heat exchanger geometry.^24^ However, in adsorption and desorption processes also the mass transfer plays a role—heat and mass transfer are strongly coupled.^25^ The concept of using driving temperature differences for both heat and mass transfer processes as presented by Wittstadt et al.^15^ allows for the calculation of an overall effective heat conductance UA of adsorption heat exchangers and evaporator-condensers.

Up to now, this concept was applied to evaluate the adsorption dynamics of small-scale samples by calculating effective heat and mass transfer resistance $R={\left(UA\right)}^{-1}$ as presented by Ammann et al.^26^ and Velte et al.^27^ Wittstadt et al.^15^ and later Velte et al.^28^ evaluated adsorption heat exchanger designs with this concept. Although it is helpful being able to compare different adsorption heat exchanger designs based on effective heat and mass transfer resistances, it is desirable to use these quantities for a prediction of heat flow rates or the widely used (volume or mass specific) cooling power (SCP).

In this research article, we combine basic heat exchanger theory and concept of effective heat and mass transfer resistances to provide a comprehensive and simple calculation of efficiency and heat flow rates of adsorption heat transformation devices. To evaluate the prediction quality of the calculation procedure, we use measurement data of two differently sized adsorption modules.

## Results

### Calculation of efficiency and heat flow rates

**Figure 1:**
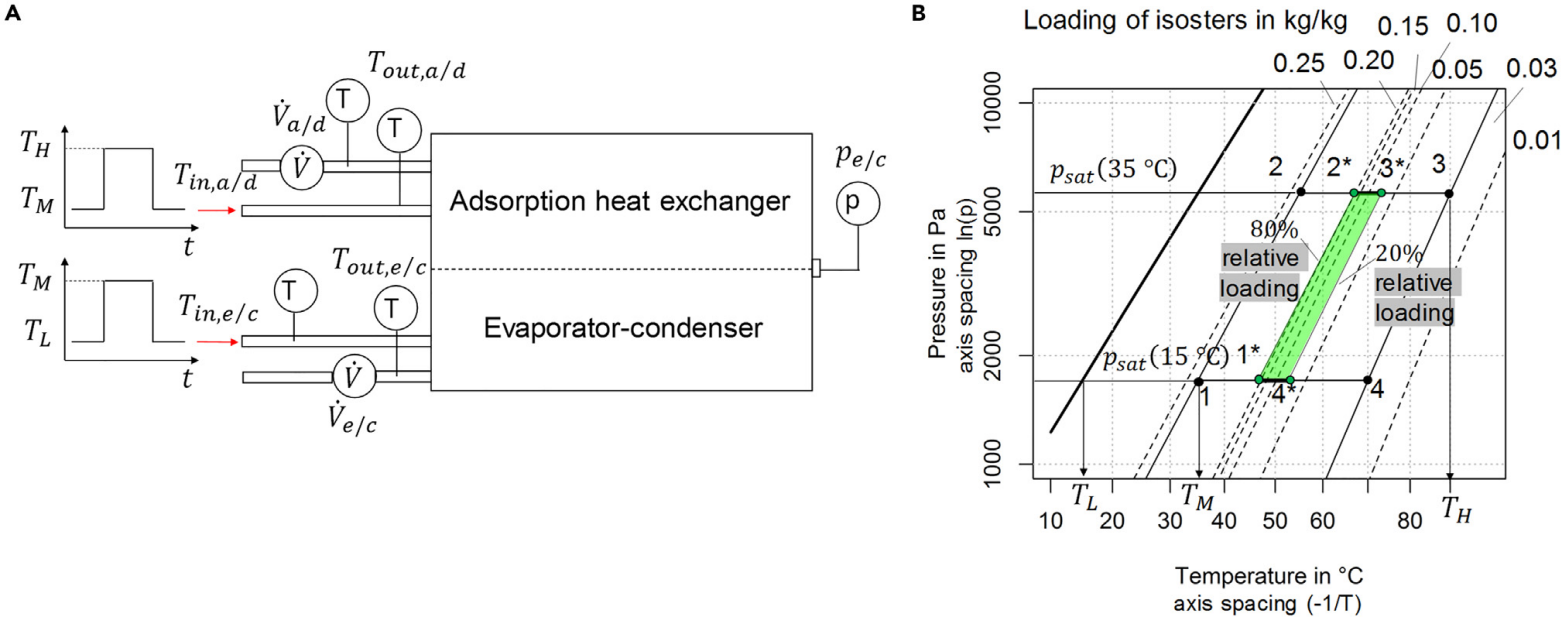
Adsorption module and ideal process (A) Schematic drawing of an adsorption module with ideal inlet temperature profiles and sensor positions. The inlet temperatures $T_{in,a/d}$ and $T_{in,e/c}$ change periodically. (B) Ideal heat pump/cooling process in the ln(p)-(-1/T)-diagram for $T_{L}=15{}^{\circ}C$, $T_{M}=35{}^{\circ}C$, $T_{H}=90{}^{\circ}C$, and SAPO-34-water working pair.

Heat transformation processes are defined as heat exchanges on the three temperature levels $T_{L}$, $T_{M}$, $T_{H}$ (low, medium, high).^14^ The inlet temperature of the adsorption heat exchanger changes periodically between $T_{M}$ (adsorption) and $T_{H}$ (desorption), whereas the evaporator-condenser inlet temperature changes periodically between $T_{L}$ (evaporation) and $T_{M}$ (condensation) as illustrated in the schematic drawing in Figure 1A. In Figure 1B, the corresponding ideal process (1→2→3→4) is illustrated in the ln(p)-(-1/T) diagram. Starting from maximum loading in point 1, the adsorption heat exchanger is heated up without changing the loading, i.e., no water is desorbed on the isostere (1→2). With increasing temperature (2→3) the desorption of water vapor starts. The pressure of the isobar (2→3) is determined by the temperature of the condenser ($T_{M}$). When reaching the minimum loading at $T_{H}|p_{sat}\left(T_{M}\right)$ in point 3, the adsorption heat exchanger is cooled down without changing the loading (3→4). With decreasing temperature, the adsorption of water vapor starts (3→4). The pressure of the isobar (3→4) is determined by the temperature of the evaporator ($T_{L})$. Since reaching the equilibrium states (points 1, 2, 3, 4) requires long half cycle times, in most practical applications the equilibrium states are never reached. Instead of reaching maximum or minimum loading, the practical process is interrupted before and the loading changes for instance from point 2∗ → 3∗ (and 4∗ → 1∗) as illustrated in Figure 1B with the green rhomb (between 20% and 80% of the equilibrium loading). In case of the SAPO-34-water working pair, it can be stated that the practically relevant adsorption and desorption processes (green rhomb) happen in a relatively narrow temperature range.

From the three temperature levels $T_{L}$, $T_{M}$, and $T_{H}$ the temperature thrust $\Delta T_{thr}$ and the temperature lift $\Delta T_{lft}$ can be calculated:

(Equation 1) $$\Delta T_{thr}=T_{H}-T_{M}$$

(Equation 2) $$\Delta T_{lft}=T_{M}-T_{L}$$

In case of adsorption heat pumps and chillers the heat transferred to the heat transfer fluid $Q_{a}$ in the adsorption heat exchanger during the adsorption half cycle consists of the following terms:

(Equation 3) $$Q_{a}=\underset{Q_{a,it}}{\underset{︸}{\Delta X\cdot M_{sorb}\cdot \Delta h_{ad}}}+\Delta T_{thr}\cdot C_{p,tot,s}-{\dot{Q}}_{loss,\mathrm{int}}\cdot t_{hc}$$

The first term of Equation 3 accounts for the adsorptive heat release, with ΔX being the loading spread, $M_{sorb}$ the mass of the dry adsorbent, and $\Delta h_{ad}$ the adsorption enthalpy. The second term accounts for the temperature change of the total thermal capacity of the adsorption heat exchanger $C_{p,tot,s}$ between $T_{H}$ and $T_{M}$. The third term accounts for the internal thermal losses between adsorption heat exchanger and combined evaporator-condenser. The calculation of ${\dot{Q}}_{loss,\mathrm{int}}$ and its experimental quantification is described in detail in Note S1 and illustrated in Figure S4. The working fluid that is adsorbed in the adsorption heat exchanger is evaporated in the evaporator-condenser. Similar to Equation 3 the heat transferred in the evaporator-condenser $Q_{e}$ consists of a term accounting for the evaporation with the evaporation enthalpy $\Delta h_{v}$, a capacitive term with the total thermal capacity of the evaporator-condenser $C_{p,tot,e}$, and a third term for the internal losses.

(Equation 4) $$Q_{e}=-\underset{Q_{e,it}}{\underset{︸}{\Delta X\cdot M_{sorb}\cdot \Delta h_{v}}}+\Delta T_{lft}\cdot C_{p,tot,e}+{\dot{Q}}_{loss,\mathrm{int}}\cdot t_{hc}$$

The thermal efficiency in case of heat pump applications is defined as the ratio of heat extracted from the sorption module during adsorption $Q_{a}$ and condensation $Q_{c}$ and heat transferred to the sorption module during desorption $Q_{d}$:

(Equation 5) $$COP_{h}=\frac{{Q_{c}+Q}_{a}}{-Q_{d}}$$

**Figure 2:**
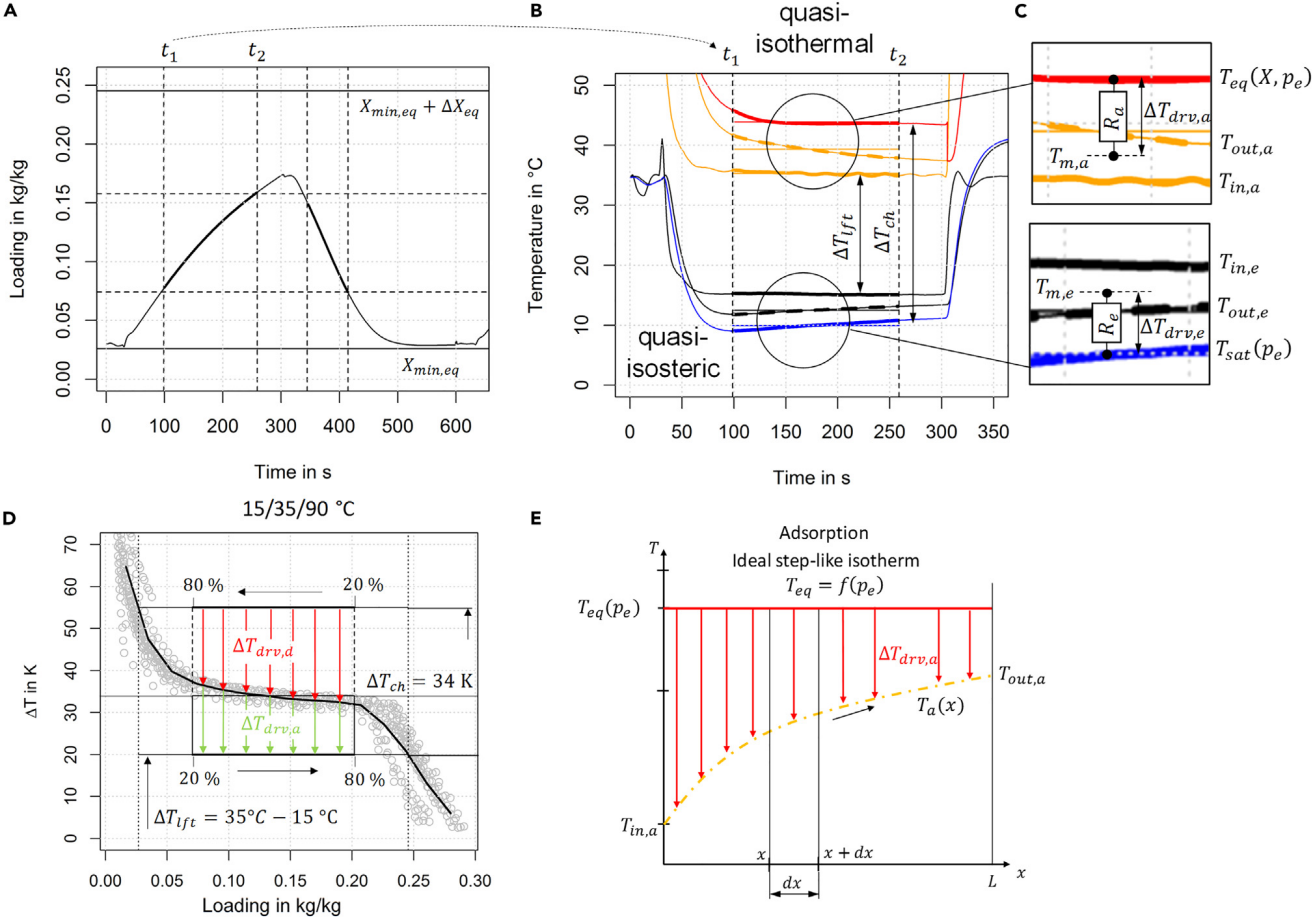
Temperature and loading curves of an adsorption module (A) Loading curve calculated out of the energy balance of the evaporator-condenser. The vertical dashed lines denote the beginning and the end of the quasi-isothermal phase of each half cycle. (B) Temperature curves for adsorption heat exchanger and evaporator-condenser; inlet and outlet temperatures are measured, saturation temperature $T_{sat}$ is calculated with the measured pressure $p_{e/c}$ in the adsorption module, and equilibrium temperature $T_{eq}$ is calculated with Equation 12 using equilibrium data. (C) Driving temperature differences $\Delta T_{drv}$ and effective heat and mass transfer resistances R in the quasi-isothermal phase. A complete characteristic temperature difference chart can be found in Figure S5 in Note S2. (D) Equilibrium data of SAPO-34-water working pair in terms of $\Delta T_{ch}$ plotted over loading X. The gray points represent equilibrium data from a small-scale sample as detailed in Note S1; the maximum and minimum loading is calculated for $T_{L}=15{}^{\circ}C$, $T_{M}=35{}^{\circ}C$, $T_{H}=90{}^{\circ}C$. The (ideal) driving temperature differences $\Delta T_{drv,a/d,\infty }$ refers to the case of an infinitely high capacity flow rate in ADHX and EC. (E) Temperature curves over the heat exchanger length of the adsorption heat exchanger in case of ideal step-like isotherm. In this case, the equilibrium temperature $T_{eq}$ (heat exchanger primary side) is constant over the heat exchanger length and depends only on the pressure $p_{e}$. The inlet and outlet temperatures refer to the heat transfer fluid (water) on the secondary heat exchanger side. The temperature curves of the evaporator-condenser can be found in Figure S6 in Note S2. Additionally, the temperature curves of the adsorption heat exchanger in case of an ideal linear isotherm are shown in Figure S7 in Note S2.

As shown in Figure 2 A/B, adsorption and desorption half cycle can be divided into a transient switching phase when the energy balance is dominated by the capacitive term in Equations 3 and 4 and a quasi-isothermal, close-to-stationary phase when the energy balance dominated by the adsorptive term. By splitting the adsorption and desorption half cycle into a quasi-isosteric switching phase and a quasi-isothermal phase, the main temperature change can be attributed to the quasi-isosteric switching phase. Within the switching phase, the heat transfer processes are clearly dominated by the capacitive term in the transient energy balance in Equations 3 and 4. However, as soon as the quasi-isosteric switching phase comes to an end, the temporal temperature gradients get smaller with

(Equation 6) $$\Delta h_{ad}\cdot \frac{dX}{dt}\cdot M_{sorb}\gg C\frac{dT}{dt}\text{,}$$

and we can assume a quasi-isothermal energy balance within the heat exchanger dominated by the adsorptive term. This is the main assumption on which the following considerations are founded. In this quasi-isothermal phase (${{\dot{Q}}_{a}\approx \dot{Q}}_{a,it}$), the heat flow rate due to the adsorptive term can be expressed as

(Equation 7) $${\dot{Q}}_{a,it}=\frac{\Delta X}{\Delta t_{it}}\cdot M_{sorb}\cdot \Delta h_{ad}=R_{a}^{-1}\cdot \Delta T_{drv,a}$$

with $R_{a}$ being the effective heat and mass transfer resistance of the adsorption heat exchanger, $\Delta t_{it}$ the duration of the quasi-isothermal phase, and $\Delta T_{drv,a}$ the driving temperature difference.

The driving temperature difference is defined in Equation 8 according to Shah et al.^23^ under the assumption of an ideal step-like isotherm, leading to the temperature profile shown in Figure 2E in the adsorption heat exchanger. A similar equation for the driving temperature difference $\Delta T_{drv,e}$ is applied for the evaporator-condenser as detailed in Note S2 and illustrated in Figure S6. The driving temperature differences and their relation to the experimentally measured temperatures are illustrated in Figure 2C.

(Equation 8) $$\Delta T_{drv,a}=\frac{T_{out,a}-T_{in,a}}{\ln\frac{T_{eq}\left({X,p}_{e}\right)-T_{in,a}}{T_{eq}\left(X,p_{e}\right)-T_{out,a}}}$$

If the half cycle time $t_{hc}$ is specified and the effective heat and mass transfer resistances $R_{a}$ and $R_{e}$ are known, the definition of the driving temperature differences $\Delta T_{drv,a}$ and $\Delta T_{drv,e}$ introduces the unknown temperatures $T_{eq}$, $T_{out,a}$, $T_{out,e}$, and $T_{sat}$. To solve this equation system, the heat exchanger effectiveness ϵ according to Shah et al.^23^ is introduced:

(Equation 9) $$\epsilon =\left\{ \begin{array}{c} \begin{array}{cc} \frac{T_{out,a/d}-T_{in,a/d}}{T_{eq}\left(X,p_{e/c}\right)-T_{in,a/d}} & \text{Adsorption}/\text{Desorption} \end{array}\\ \begin{array}{cc} \frac{T_{out,e/c}-T_{in,e/c}}{T_{sat}\left(p_{e/c}\right)-T_{in,e/c}} & \text{Evaporation}/\text{Condensation} \end{array} \end{array}\right.$$

The heat exchanger effectiveness ϵ in Equation 9 is calculated with inlet and outlet temperatures of the components $T_{in}$, $T_{out}$, and the equilibrium temperature $T_{eq}$ in case of the ADHX in adsorption or desorption and the saturation temperature $T_{sat}$ in case of the EC in evaporation or condensation.

The number of transfer units (NTU) is defined as the ratio of the reciprocal effective heat and mass transfer resistance and the capacity flow rate of the heat exchanger according to Shah et al.^23^

(Equation 10) $$NTU=\frac{(U{A)}_{tot}}{{\dot{C}}_{HTF}}=\frac{R^{-1}}{{\dot{C}}_{HTF}}$$

By specifying the mass flow rate through the adsorption heat exchanger and the evaporator-condenser, the capacity flow rate is known. Thus, the ε-NTU relationship in Equation 11 yields two equations for the four unknown temperatures. The ε-NTU relationship is valid for evaporation or condensation^23^ and under the assumption of an ideal step-like isotherm leading to the temperature profile shown in Figure 2E also for adsorption or desorption.

Equation (11) ϵ=1−exp(−NTU)

Adsorption equilibrium data are usually given in the form of loading X(p,T). From this, the equilibrium temperature $T_{eq}\left(X,p\right)$ is defined as the temperature, at which a certain loading X at a certain pressure p is reached. In Equations 8 and 9, the saturation temperature $T_{sat}$ and the equilibrium temperature $T_{eq}\left(X,p\right)$ are needed. We now define the "characteristic temperature difference" $\Delta T_{ch}\left(X,p\right)$ for a given working pair as the difference between the equilibrium temperature $T_{eq}\left(X,p\right)$ and the saturation temperature $T_{sat}\left(p\right)$ of the pure vapor at the respective pressure in Equation 12 as suggested by Laurenz^8^:

(Equation 12) $$\Delta T_{ch}\left(X,p\right)=T_{eq}\left(X,p\right)-T_{sat}\left(p\right)$$

This characteristic temperature difference $\Delta T_{ch}\left(X,p\right)$ can be calculated from any equilibrium dataset X(p,T). Practically, $\Delta T_{ch}$ is the temperature difference between an ADHX and an EC in one housing (same pressure, cf. Figure 1) and both perfectly insulated (no heat or mass flow), i.e., the adsorption-thermodynamic equivalent to the open circuit voltage of a battery cell.

As illustrated in Figure 2D, the pressure dependency of $\Delta T_{ch}\left(X,p\right)$ is small, thus, for engineering purposes, different isotherms and isobars maybe represented by a single curve $\Delta T_{ch}\left(X\right)$. For adsorbents with a step-like isotherm, the characteristic temperature difference $\Delta T_{ch}\left(X,p\right)$ can be further simplified to a constant mean value over a wide loading range as illustrated in Figure 2D. In the following, a constant value of 34 K is taken for all calculations as “characteristic” for SAPO-34-water.

The last equation necessary for the calculation results out of the mass balance in the quasi-isothermal phase. The working fluid that is adsorbed in the adsorption heat exchanger must be evaporated in the evaporator-condenser. Finally, the algebraic equation system can be solved with the closing condition

(Equation 13) $$\frac{{\dot{Q}}_{a,it}}{{\dot{Q}}_{e,it}}=\frac{\Delta h_{ad}}{\Delta h_{v}}$$

by using an algorithm for zero-point search.

For calculating the amounts of heat $Q_{a}$ and $Q_{e}$ in Equations 3 and 4, a relationship between ΔX and $t_{hc}$ is required that can be found in Note S3 as illustrated in Figures S8 and S9.

### Application to the rating problem

The most relevant key performance indicators for practical purposes are efficiency and mean heat flow rate. With values for the effective heat and mass transfer resistances of the EC and the ADHX, inlet temperatures, and mass flow rates, the mean heat flow rates in the adsorption, desorption, evaporation, and condensation are calculated according to Equations 3 and 4. The experimental data of two adsorption modules as described in Note S1 (Figure S1; Table S2; Figure S2, “Size S” and “Size L” module) serves as a reference to evaluate the prediction quality of the calculation. The test rig as shown in Figure S3 in Note S1 allows for a fast switching (<30 s from one stable [±0.5 K] temperature level to the other) between adsorption and desorption (and consequently evaporation and condensation) temperature levels, ensuring that the measurement reflects the dynamics of the heat and mass transfer process of the adsorption module rather than the dynamics of the test rig.

**Figure 3:**
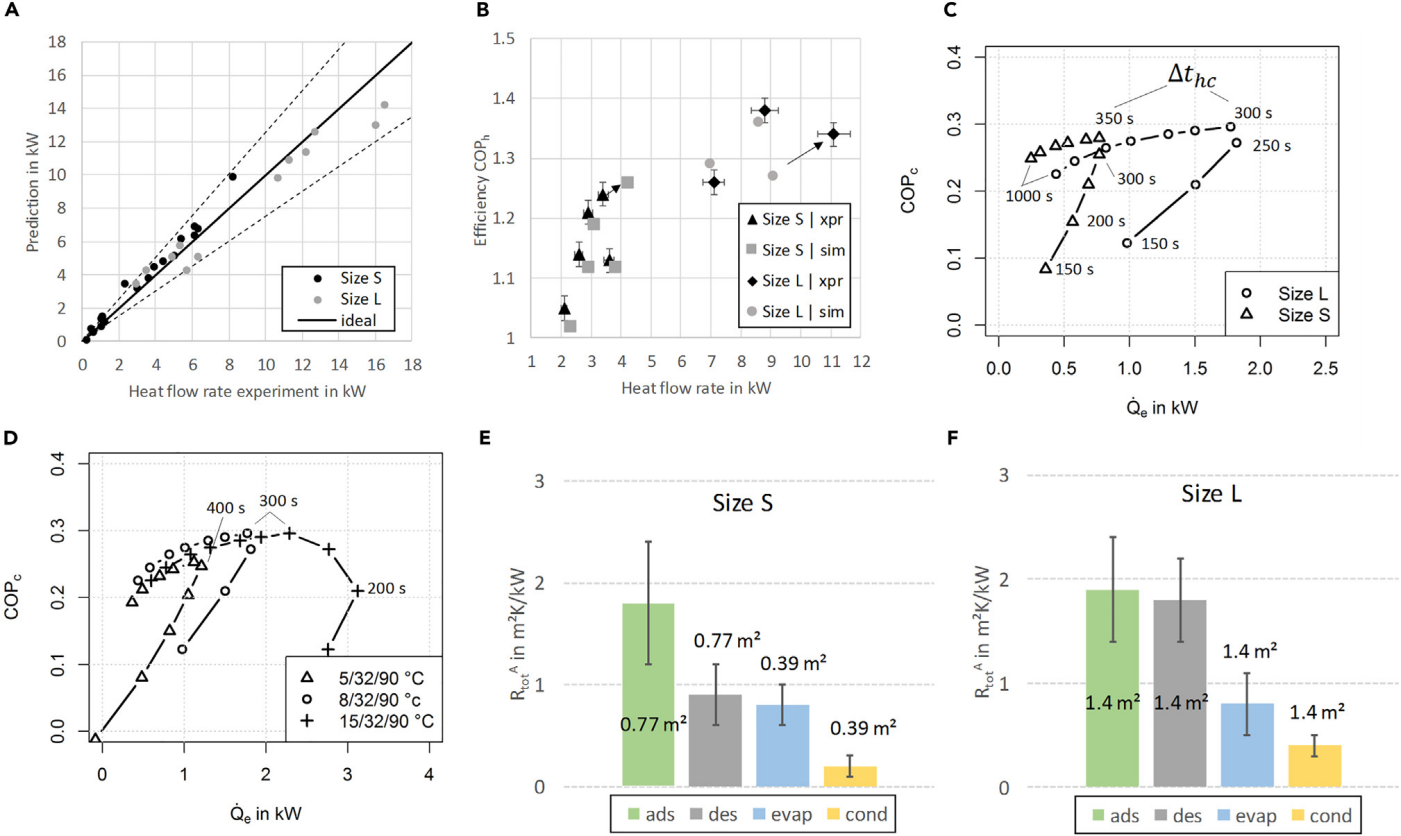
Prediction quality and performance evaluation (A) Predicted heat flow rates compared with experiment. The diagonal (“ideal”) shows the ideal model prediction quality, i.e., calculated value is equal to experimental value. In this case all points would collapse to this line. (B) Comparison of prediction and experiment in terms of efficiency and heat flow rate (heating application); see also Figure S14 and Table S4 in Note S6. Error bars of the experimental data points are calculated with Gaussian error propagation method. (C) Performance evaluation of “Size L” and “Size S” adsorption modules under the same boundary conditions as listed in Table 1 (cooling application). The times shown in this figure are half cycle times. (D) Dependency of efficiency and cooling power on half cycle times and temperature boundary conditions (“Size L” module). The times shown in this figure are half cycle times. (E) Effective heat and mass transfer resistances scaled with heat exchanger primary area in adsorption (ads), desorption (des), evaporation (evap), and condensation (cond) of “Size S” adsorption module. Error bars are calculated with Gaussian error propagation method. (F) Effective heat and mass transfer resistances scaled with heat exchanger primary area in adsorption (ads), desorption (des), evaporation (evap), and condensation (cond) of “Size L” adsorption module. Error bars are calculated with Gaussian error propagation method.

One measurement of each adsorption module (xpr1_L and xpr1_S as listed in Table S3 in Note S1) is used to calculate the effective heat and mass transfer resistances of the evaporator-condenser and the adsorption heat exchanger (calibration), resulting in the values as shown in Figures 3E and 3F. The other measurements serve as validation points. For more details the reader is referred to Note S4. A comparison of experimentally measured and calculated values for efficiency and mean heat flow rates is shown in Figures 3A and 3B. The prediction quality of the calculated heat flow rates (in adsorption, desorption, evaporation, and condensation) in Figure 3A is evaluated with root-mean-square deviation (RMSD) and coefficient of variance (CV). The RMSD for “Size L” experiments is ±1.31 kW, leading to a CV of 15%. For “Size S” experiments the RMSD is ±0.6 kW, resulting in a CV of 19%. The CV of the heat flow rate relevant for the application (here: adsorption and condensation) shown in Figure 3B is 13% for “Size L” experiments and 14% for “Size S” experiments. The prediction quality of the efficiency as shown in Figure 3B is also evaluated with RMSD and CV. In case of “Size L” experiments, the RMSD is 0.05 and the CV is 3%, whereas the “Size S” experiments are predicted with an RMSD of 0.02 and a CV of 2%. A more detailed analysis reveals that the predicted efficiency is within ±0.03 (absolute) range of the values obtained in the experiments except for one outlier in case of “Size L” experiments.

Based on this experimentally validated calculation, the performance of the “Size S” module and the “Size L” module can be compared under the same temperature boundary conditions and same NTU. For this comparison the cooling application is chosen. The inlet temperatures and mass flow rates are listed in Table 1. The effective heat and mass transfer resistances of the EC and the ADHX for this calculation are taken according to Figures 3E and 3F.

**Table 1:** Boundary conditions for calculation of efficiency and heat flow rates of “Size S” and “Size L” module

Parameter	Unit	Size S	Size L
Inlet temperature $T_{HT,in}$	°C	8	8
Inlet temperature $T_{MT,in}$	°C	32	32
Inlet temperature $T_{LT,in}$	°C	90	90
Mass flow rate ADHX ${\dot{M}}_{HTF,s}$	kg/s	0.26	0.43
Mass flow rate EC ${\dot{M}}_{HTF,e}$	kg/s	0.14	0.50
NTU ADHX	1	0.4	0.4
NTU EC	1	0.8	0.8

The results in terms of efficiency and heat flow rate (cooling application) are shown in Figure 3C. Following the curves beginning with short half cycle times (150 s), efficiency $COP_{c}$ and heat flow rate ${\dot{Q}}_{e}$ increase with the half cycle time until a maximum is reached. Longer half cycle times beyond this maximum lead to lower efficiencies due to the internal heat losses between ADHX and EC. Optimal half cycle times lie between 250 and 300 s for the “Size L” module and 300 and 350 s for the “Size S” module. This can be explained with the fact that “Size S” and “Size L” adsorption modules have a different adsorbent (dry) mass $M_{sorb}$ and different effective heat and mass transfer resistances $R_{s/e}$. According to Equation S16 in Note S3, these quantities change the slope of the gradient ΔX/Δt. Thus, the half cycle time to achieve the same loading spread ΔX changes if the sorption modules have different properties. As another result of Equation S16 in Note S3, the driving temperature difference $\Delta T_{drv}$ as well has an impact on the half cycle time.

This dependency is further explored as illustrated in Figure 3D. In case of 5/32/90°C and 8/32/90°C temperature boundary conditions, there is an optimum of efficiency and cooling power at half cycle times of 400 s and 300 s, respectively. In case of the 15/32/90°C temperature boundary condition, two optima exist: efficiency optimum for 300 s half cycle time and cooling power optimum for 200 s half cycle time. According to Equation S16 in Note S3, an increasing driving temperature difference $\Delta T_{drv}$ leads to a steeper slope of the gradient ΔX/Δt. In the calculations to generate data for Figure 3D, the ADHX properties ($M_{sorb}$, $R_{s}$, $C_{p,tot,s}$) do not change, neither does the adsorption enthalpy $\Delta h_{ad}$. Thus, to achieve the same loading spread ΔX, an increasing driving temperature difference $\Delta T_{drv}$ leads to shorter half cycle times. The driving temperature difference itself is affected by the mass flow rate (or NTU) and temperature boundary conditions as illustrated in Figure 2D. Thus, the optimal half cycle time must be chosen according to these conditions.

The other aspect in Figure 3D is the impact of the temperature lift $\Delta T_{lft,in}$ on the cooling power: with $\Delta T_{lft,in}$ of 17 K (15/32/90°C) a maximum cooling power of 3 kW can be achieved. With $\Delta T_{lft,in}$ of 27 K (5/32/90°C) the maximum cooling power drops to 1.1 kW. This observation can be explained with Equation 7—the cooling power is directly linked to the driving temperature difference in the adsorption half cycle, which increases with decreasing temperature lift $\Delta T_{lft,in}$.

### Application to the sizing problem

Sizing an adsorption module requires the sizing of the two components ADHX and EC (or evaporator and condenser in case of separate components). The sizing process yields the required heat and mass transfer resistances $R_{a}$, $R_{d}$, $R_{e}$, and $R_{c}$. As shown in Figures 3E and 3F, the heat and mass transfer resistances that are scaled with the heat exchanger primary area are nearly the same for heat exchangers with different sizes in adsorption and evaporation. These two resistances dominate the process if $\Delta {\bar{T}}_{ch}-\Delta T_{lft}<\Delta T_{thr}-\Delta {\bar{T}}_{ch}$, $R_{a}>R_{d}$, and $R_{e}>R_{c}$. Thus, the required heat exchanger primary areas of the ADHX $A_{s}$ and the EC $A_{e}$ can be calculated with Equations 14 and 15, respectively. The relationships between overall thermal capacity $C_{p,tot}$ and $R_{a}$ as well as adsorbent mass $M_{sorb}$ and $R_{a}$ are illustrated in Figure S12 and S13, respectively.

(Equation 14) $$A_{s}=\max\left(\frac{R_{s,ads}^{A}}{R_{s,ads}};\frac{R_{s,des}^{A}}{R_{s,des}}\right)$$

(Equation 15) $$A_{e}=\max\left(\frac{R_{e,ads}^{A}}{R_{e,ads}};\frac{R_{e,des}^{A}}{R_{e,des}}\right)$$

**Figure 4:**
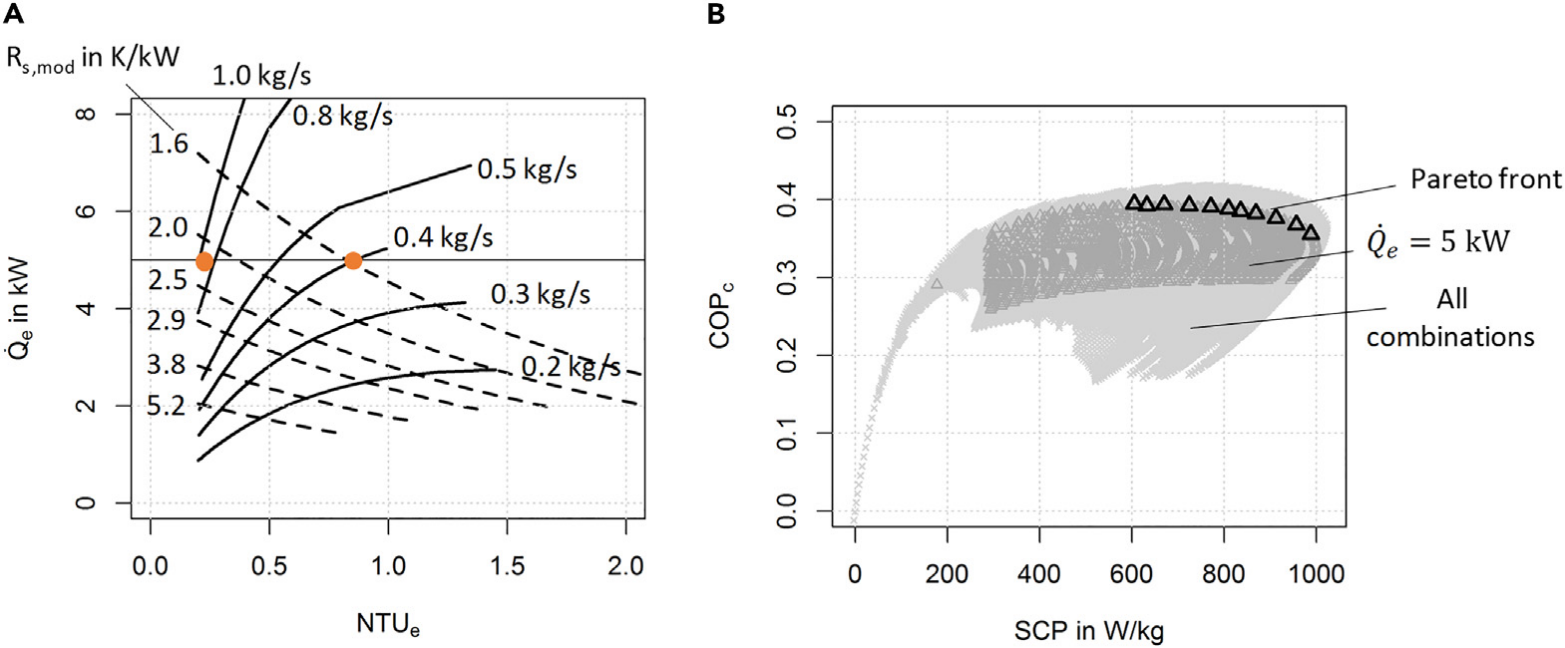
Sizing problem for cooling application at $T_{L}=8{}^{\circ}C$, $T_{M}=30{}^{\circ}C$, and $T_{H}=80{}^{\circ}C$ (A) Heat flow rate in the evaporation phase (cooling power) plotted against the NTU. Isolines are plotted for constant mass flow rates and for constant overall heat and mass transfer of the module $R_{\mathrm{mod}}$. (B) Efficiency (cooling application and adsorbent mass) plotted against specific cooling power SCP of all calculated cases.

The details of the sizing process calculation are described in Note S5 and illustrated in Figure S11. The output of the calculation for the temperature triple 10/30/80°C is shown in Figure 4A in terms of heat flow rate of the EC in the evaporation phase (cooling power) plotted over the NTU of this component. For design choices two different isolines are plotted: lines of constant overall heat and mass transfer resistance of the module $R_{\mathrm{mod}}$ according to Equation S20 in Note S4 and lines of constant mass flow rates through ADHX and EC. Following the lines of constant mass flow rate, it is obvious that the cooling power increases with the component size (i.e., $R_{\mathrm{mod}}$ decreases). In contrast, following the lines with constant $R_{\mathrm{mod}}$, the cooling power decreases with the mass flow rate. If we set the desired cooling power to 5 kW, a design range between 0.3 kg/s and 0.8 kg/s mass flow rate and 1.6 K/kW and 2.5 K/kW overall heat and mass transfer resistance can be identified.

For a meaningful choice of the basic design parameters, we must include other performance indicators like efficiency and specific cooling power. The dependency between these two performance indicators is shown in Figure 4B.

The most important parameters of the design variants on the Pareto front are listed in Table 2. The range of the overall effective heat and mass transfer $R_{\mathrm{mod}}$ resistance is relatively narrow—between 1.7 K/kW and 2.3 K/kW like $R_{a}$ that is also within a narrow range between 0.6 K/kW and 1 K/kW. However, within this range a broad spectrum of the combination of the component sizes can be found—#1 with a large ADHX and a small EC (γ=0.5) or #11 with a rather small ADHX. To achieve a high efficiency and high specific cooling power, the required NTU of the components is in nearly all Pareto optimal points at its defined minimum of 0.2—the intensification of the heat and mass transfer processes requires a high-capacity flow rate. With increasing specific cooling power and decreasing size of the ADHX as shown in Table 2, the efficiency is decreasing with increasing SCP. This can be explained with the larger size of the EC (γ increases)—the increasing thermal mass of the EC lowers the efficiency.

**Table 2:** Design parameters of Pareto optimal design variants, ${\dot{Q}}_{e}$ is 5 kW, 10°C evaporator inlet, and 30°C adsorption heat exchanger inlet

#	$R_{a}$ in K/kW	γ	$R_{\mathrm{mod}}$ in K/kW	$NTU_{s}$	$NTU_{e}$	$M_{sorb}$	SCP in W/kg
1	0.6	0.5	2.3	0.2	0.2	8.1	609
2	0.7	0.6	2.1	0.4	0.2	7.7	641
3	0.7	0.6	2.2	0.2	0.2	7.4	675
4	0.7	0.7	2.1	0.2	0.2	6.9	730
5	0.8	0.8	2	0.3	0.2	6.6	760
6	0.8	0.9	2	0.2	0.2	6.4	797
7	0.9	1	2	0.2	0.2	6	848
8	0.9	1.1	1.9	0.2	0.2	5.8	876
9	0.9	1.3	1.8	0.2	0.3	5.6	908
10	1	1.5	1.8	0.2	0.2	5.3	951
11	1	1.9	1.7	0.2	0.2	5.1	990

The final design choice might involve factors that are not part of our study like costs, minimizing electrical power consumption required for pumps, limitations by component availability and manufacturing, and mechanical stability.

## Discussion

The prediction quality of efficiency and heat flow rates by the calculation proposed in this research paper strongly depends on the choice of the values for the effective heat and mass transfer resistances. In this study, the simplest choice of constant effective heat and mass transfer resistances was made. Compared with a detailed transient numerical model the prediction quality of efficiency and heat flow rate is lower as discussed in Note S6 (Figure S14; Table S4). The reason for this discrepancy can be found in the dependency of diffusion coefficients on temperature and loading as the results of Laurenz et al.^29^ show. The superior prediction quality of the detailed transient numerical model shown in Note S6 has its reason in the variation of the adsorbate diffusion coefficient to match the experimental data. Thus, if the effective heat and mass transfer resistance is dominated by a temperature- and loading-dependent diffusion coefficient, this leads to the necessity to adapt the effective heat and mass transfer resistance to match this dependency. More experiments with different temperature boundary conditions would have to be performed to learn more about such a dependency. However, considering the simplicity of the calculation and the use of temperature and loading independent effective heat and mass transfer resistances, the prediction quality is sufficient for first-order design decisions.

For such first-order design decisions the available data are often only limited. If there are no experimental data on a similar design, no effective heat and mass transfer resistances can be calculated. One way to get first-order estimates for effective heat and mass transfer resistances is to use data gained from small-scale samples. For example, the area-scaled effective heat and mass transfer resistance $R_{a}$ of two small-scale samples shown in Figure S10 in Note S4 is around 1.3 m^2^K/kW on average. This is quite close to the values of the much larger ADHX as shown in Figures 3E and 3F, leading to the conclusion that the data from small-scale samples can be used as a good starting point for ADHX design. For further information on the small-scale samples, the reader is referred to Figure S1 and Table S1 in Note S1. In case of the EC, effective heat and mass transfer resistances can be obtained from evaluation of evaporation (or condensation) experiments that are available in literature. For instance, values for a variety of plain and finned tubes are provided by Lanzerath et al.^30^ and Seiler et al.^31^; values for a tube-fin heat exchanger are provided by Volmer et al.^32^ More recently, Mikhaeil et al.^33^ provided values for asymmetric plate heat exchangers. Further, effective heat and mass transfer resistances may also be estimated directly from basic transport and geometry parameters as proposed by Laurenz^8^ for a set of typical heat and mass transfer mechanisms in adsorption heat exchangers.

The computational costs of the proposed calculation with basic adsorption heat exchanger theory are very low compared with an experiment or a calculation with a transient numerical model. Generating one data point in Figure 4B requires the calculation of approximately 50 variables, which takes <0.1 s on a computer with Intel Core i5-8365SU CPU @ 1.60 GHz and 16.0 GB RAM. The calculation with a more complicated, transient numerical model requires to solve approximately 20,000 equations with an average step size of 0.5 s, resulting in a dataset with 144·10^6^ scalars, which takes around 5 s on the same computer. The experimental measurement requires the recording of at least 8 variables at a minimum frequency of 2 Hz, resulting in a dataset with 57,600 scalars if the measurement duration is 1 h. This computationally very inexpensive calculation of efficiency and heat flow rates with basic adsorption heat exchanger theory as presented in this research article allows for quick first-order design decisions as well as the migration of the code on a microcontroller. Together with an appropriate model predictive control framework, the half cycle time for reaching the optimal efficiency for a desired heat flow rate can be calculated in the real-world application.

### Limitations of the study

A transient numerical model usually includes following parts.

(1) Differential equations accounting for heat and mass transfer phenomena
(2) Thermal mass of the heat exchangers
(3) Equilibrium model to calculate equilibrium temperature and/or equilibrium pressure
(4) Model for the heat transfer fluid that calculates the temperature difference between inlet and outlet depending on heat flow rate and volume flow rate
(5) Calculation of internal losses

The proposed calculation method includes all these parts, yet not spatially discretized and with simplifications that are discussed in the following. The heat and mass transfer phenomena are considered with constant values for effective heat and mass transfer resistances. It is known from other studies^29^^,^^34^ that diffusion coefficients vary with temperature and loading. Thus, the mass transfer resistance will change with temperature boundary conditions and loading and consequently vary within the half cycle. As the method is presented here, this dependency is neglected, lowering the prediction quality for temperature boundary conditions and half cycle times that are not nearby the calibration points. It must be added that information on the temperature and loading dependency of diffusion coefficients is quite limited. Thus, even if it is possible to implement such dependencies in a more complicated, transient numerical model it is often not done due to this lack of data. Consequently, this issue also lowers the prediction quality of a transient numerical model.

The thermal mass of the heat exchangers is included as well as the calculation of internal losses. The equilibrium temperatures are calculated with the method of the mean characteristic temperature difference, which can be regarded as a radically simplified equilibrium model. The temperature difference between inlet and outlet temperature is calculated with the eps-NTU method from basic heat exchanger theory that accounts for the heat flow rate and volume flow rate in the heat exchanger.

All these simplifications are based on the assumption of constant inlet temperatures throughout the half cycle that are necessary to achieve a “quasi-isothermal” phase. Varying inlet temperatures within the half cycle as they can be observed in case of constant heat flow rate processes (e.g., heating up the ADHX with a gas burner of constant power) challenge this assumption and are not covered by the proposed calculation method as presented here. Thus, the proposed calculation method is limited to processes with constant inlet temperatures as they are common for the typical operation of adsorption chillers. Of course, precise prediction of highly dynamic processes (e.g., heat recovery by delayed switching of return valves) requires a transient numerical model.

The applicability of the proposed calculation method as presented here is also limited to working pairs having a step-like isotherm that allows for the calculation of a constant mean value of the characteristic temperature difference. This makes this method best suited for a broad range of physisorption working pairs (SAPO-34, CAU-10-H, aluminum fumarate with water as working fluid) as well as chemisorption working pairs that usually have sharp steps instead of just a “step-like” isotherm. However, the limitation to step-like isotherms excludes silica gel as well as hydrophilic zeolites (13X, NaY). This said, it can be added that our ongoing research and development of the calculation method shows that this limitation can be overcome with some modifications that will be published in the future.

## STAR★Methods

### Key resources table

**Table:** 

REAGENT or RESOURCE	SOURCE	IDENTIFIER
**Deposited data**		

Experimental results adsorption module ‘Size S’	Velte et al.^28^	https://dx.doi.org/10.3390/en15082823
Experimental results adsorption module ‘Size L’	Wittstadt et al.^15^	https://dx.doi.org/10.1016/j.renene.2016.08.061
Experimental results small-scale samples SSc-1, SSc-2	Velte^6^	https://dx.doi.org/10.6094/UNIFR/154691
Numerical results (detailed model) adsorption module ‘Size L’	Velte^6^	https://dx.doi.org/10.6094/UNIFR/154691
Equilibrium data for SAPO-34-water working pair	Velte et al.,^28^ this paper	https://dx.doi.org/10.3390/en15082823, https://dx.doi.org/10.24406/fordatis/262

**Software and algorithms**		

Original code for performance prediction using basic adsorption heat exchanger theory	This paper	https://dx.doi.org/10.24406/fordatis/262
R: A Language and Environment for Statistical Computing	R Foundation for Statistical Computing	https://www.R-project.org/

**Other**		

Adsorption module ‘Size S’	Fraunhofer ISE	N/A
Adsorption module ‘Size L’	Fahrenheit GmbH	N/A
Small-scale sample SSc-1	Fraunhofer ISE	ADOSO-0-1-012
Small-scale sample SSc-1	Fraunhofer ISE	ADOSO-0-1-025

### Resource availability

#### Lead contact

Further information and requests for resources should be directed to and will be fulfilled by the Lead Contact, Andreas Velte-Schäfer (andreas.velte-schaefer@ise.fraunhofer.de).

#### Materials availability

This study did not generate new unique reagents.

#### Data and code availability

• This paper analyzes existing, publicly available data. These accession numbers for the datasets are listed in the key resources table.
• All original code has been deposited at the Research Data Repository of Fraunhofer-Gesellschaft (Fordatis) and is publicly available as of the date of publication. DOIs are listed in the key resources table.
• Any additional information required to reanalyze the data reported in this paper is available from the lead contact upon request.

### Method details

#### General

The transient temperature curves during adsorption, desorption, evaporation, and condensation are simplified by dividing the half cycles into a highly transient switching phase and a quasi-isothermal phase. The temperatures during the quasi-isothermal phase are given as arithmetic mean values of the half cycle.

#### Calculation of efficiency and heat flow rates

Efficiency and heat flow rates are calculated the 1^st^ and the 2^nd^ law of thermodynamics and applying them to the switching phase and the quasi-isothermal phase separately. The heat flow rates are calculated by dividing the driving temperature difference with the thermal resistance. The thermal resistance is interpreted as an effective heat and mass transfer resistance that includes all relevant heat and mass transfer processes. The driving temperature difference in the adsorption heat exchanger is calculated with equilibrium data of the working pair (adsorbent and working fluid) that is transformed into the characteristic temperature difference representation. All equations needed for the calculation are described in detail in the main text and the supplemental material.

## Supplemental information

Document S1. Figures S1‒S14, Tables S1‒S4, and Notes S1–S6
