# Supplementary material for: Basic adsorption heat exchanger theory for performance prediction of adsorption heat pumps
Source: iScience. 2023 Nov 11;26(12):108432. doi: 10.1016/j.isci.2023.108432 (PMC10708995; doi:10.1016/j.isci.2023.108432)
Supplement: Document S1. Figures S1‒S14, Tables S1‒S4, and Notes S1–S6 [file mmc1.pdf]

iScience, Volume *26*

Supplemental information

Basic adsorption heat exchanger theory

for performance prediction of adsorption heat pumps

Andreas Velte-Schäfer, Eric Laurenz, and Gerrit Füldner

SUPPLEMENTAL INFORMATION

Supplemental Note 1: Main Data of Small-Scale Samples and Adsorption Modules

Pictures and schematic details of the samples used in this study are shown in Figure S1. The small-scale samples consist of a fibrous structure that is sintered on an aluminum support plate. The samples are coated with the zeotype material silico-alumino-phosphate-34 (SAPO-34) with the partial support transformation (PST) technique presented by Bauer et al.[1]. Further information on small-scale samples can be found in an earlier publication of Velte et al.[2] The main sample data is listed in Table S1.

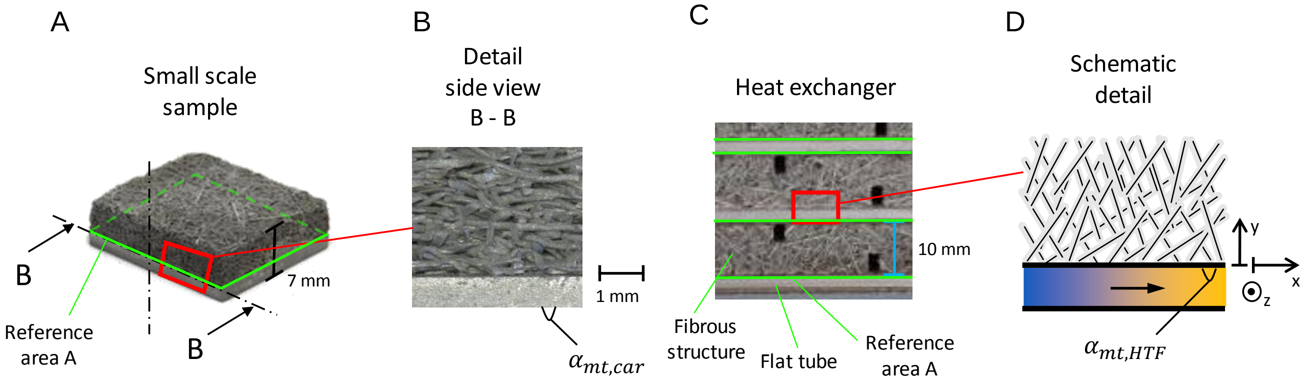

Figure S1. Fibrous structures related to Figure 1A

(A) Small scale sample.
(B) Detailed side view of small-scale sample.
(C) Adsorption heat exchanger side view.
(D) Schematic detail of adsorption heat exchanger.

Table S1. Main data of small-scale samples SSc-1 and SSc-2, related to Discussion section.

Quantity	SSc-1	SSc-2
Adsorbent mass (dry) in g	0.83	1.21
Size of the fibrous structure LxWxH in mm	35.4x20.1x2.9	22.5x20.1x5
Overall sample size including support plate LxWxH in mm	35.4x20.1x4.9	22.5x20x7
Overall heat capacity in J/K	5.2	4.8
Reference area in cm²	7.1	4.5

The adsorption modules contain an adsorption heat exchanger (ADHX) and an evaporator-condenser (EC) as shown schematically in Figure 1A in the main research article. Flat tube heat exchangers are manufactured with fibrous structures brazed in between the flat tubes. Like the small-scale samples, also the adsorption heat exchangers are coated with SAPO-34 with the PST technique. The main data of the adsorption modules is listed in Table S2, in Figure S2 the two modules with their main dimensions are shown.

Table S2. Main data of adsorption modules Size L and Size S, related to Figure 3

Quantity	Size L	Size S
**Adsorption heat exchanger**		
Adsorbent mass in kg	3.3 ± 0.3	1.5 ± 0.2
Heat exchanger dimensions with headers<br>w/o headers in mm	700 × 313 × 45<br>600 × 313 × 45	450 × 185 × 80<br>400 × 185 × 80
Volume with headers in dm³	9.9 ± 0.1	5.7 ± 0.1
Overall heat capacity in kJ/K	18.2	13.7
Reference area in m²	1.41	0.77
**Evaporator–condenser**		
Heat exchanger primary area in m²	43 ± 2	14 ± 1
Heat exchanger dimensions with headers<br>w/o headers in mm	700 × 313 × 45<br>600 × 313 × 45	450 × 158 × 45<br>400 × 158 × 45
Volume with headers in dm³	9.9 ± 0.1	3.2 ± 0.1
Overall thermal capacity in kJ/K	17.5	7
Reference area in m²	1.41	0.39
**Module**		
Dimensions (w/o insulation) in mm	730 × 320 × 147	474 × 169 × 188
Volume (w/o insulation) in dm³	34 ± 0.2	15 ± 0.2
Overall heat conductance between adsorption heat exchanger and evaporator-condenser $(UA)_{ADHX,EC}$ in W/K	8.5±2	5±2

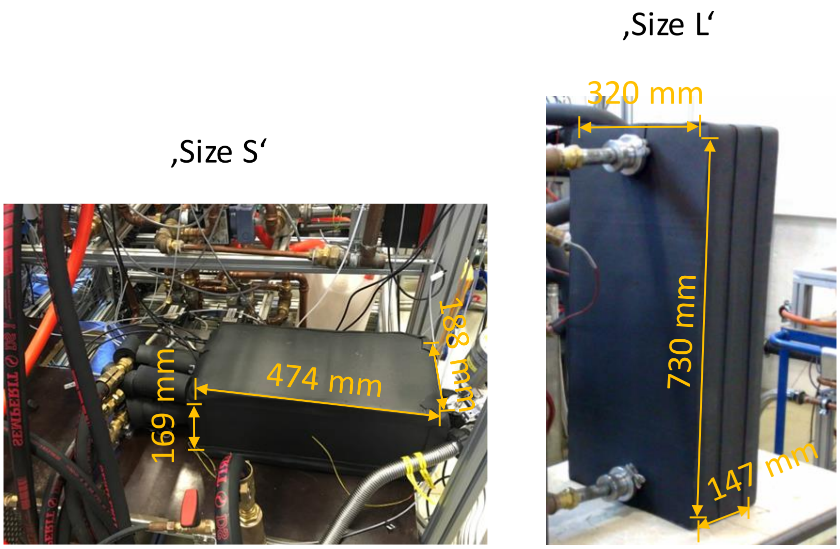

Figure S2. Main dimensions of 'Size S' and 'Size L' adsorption modules, related to Figure 3.

Values of main dimensions from Table S2 without insulation. The picture of the 'Size L' adsorption module is adapted from Wittstadt et al.[3].

The experimental data shown in Figure 3 A/B in the main research article are gathered with the experimental set-up as shown in Figure S3. The inlet temperature of ADHX and EC can be switched from T_H to T_M and T_M to T_L and vice versa. Inlet and outlet temperatures, volume flow rates, and the module pressure are recorded (with a frequency of 10 Hz). The three 500 liter tanks provide enough buffer, such that the desired inlet temperatures can be kept stable throughout the half cycle (±0.5 K). More detailed information can be found in a previous publication[4].

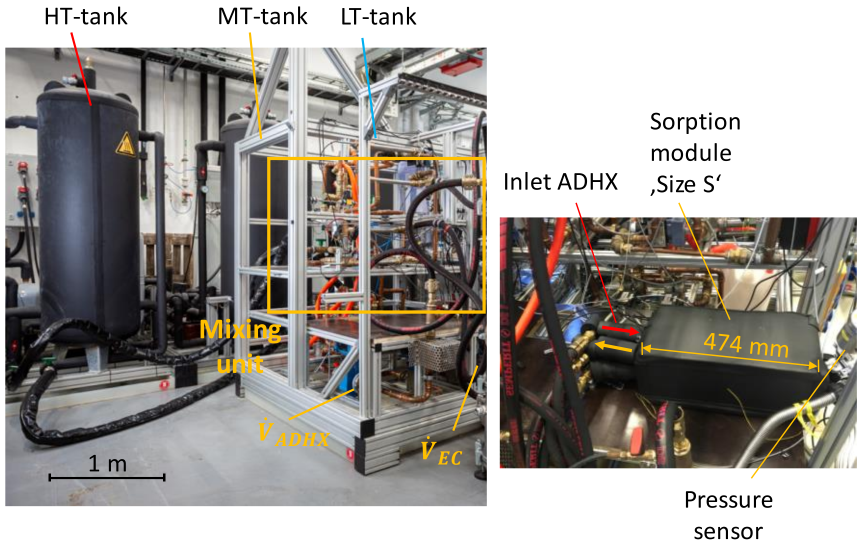

Figure S3. Sorption module test rig, related to Results section.

The boundary conditions of the experiments of 'Size S' and 'Size L' module are listed in Table S3.

Table S3. List of measurement conditions for sorption modules 'Size S' and 'Size L', related to Figure 3.

Abbreviation	Module	Inlet temperature conditions $T_L/T_M/T_H$ in °C	Half cycle time ads/des in s	Mass flow rate ADHX ads/des in kg/s	Mass flow rate EC ads/des in kg/s
xpr1_L	Size L	15/35/90	300/300	0.49 / 0.45	0.52 / 0.36
xpr2_L	Size L	19/27/85	300/300	0.49 / 0.45	0.52 / 0.36
xpr3_L	Size L	19/27/85	200/200	0.53 / 0.47	0.51 / 0.33
xpr1_S	Size S	15/35/90	300/300	0.16 / 0.16	0.17 / 0.17
xpr2_S	Size S	12/32/90	200/200	0.16 / 0.16	0.17 / 0.17
xpr3_S	Size S	5/35/95	400/200	0.16 / 0.16	0.17 / 0.17
xpr4_S	Size S	7/35/95	400/200	0.16 / 0.16	0.17 / 0.17
xpr5_S	Size S	10/35/95	400/200	0.16 / 0.16	0.17 / 0.17
xpr6_S	Size S	15/35/95	400/200	0.16 / 0.16	0.17 / 0.17

Since ADHX and EC are assembled in a single vacuum chamber an undesired heat flow between the components occurs due to radiation, conduction, and convection (water vapour). The mean heat flow rate $\dot{Q}_{loss,int}$ is calculated with Equation (S1)

$$\dot{Q}_{loss,int} = (UA)_{ADHX,EC} \cdot \left(T_{out,a/d} - T_{out,e/c}\right) \quad \text{Equation (S1)}$$

The overall heat conductance $(UA)_{ADHX,EC}$ can be quantified experimentally by integrating the component heat flow rates as shown in Figure S4. The integrated heat flow rate of the ADHX is increasing from cycle to cycle and the integrated heat flow rate of the EC is decreasing from cycle to cycle at a value of 0.31 kW in case of the 'xpr3_L' measurement. The analysis of the inlet and outlet temperatures shows that the mean temperature difference between ADHX and EC inlet temperatures is 34.5 K and the outlet temperatures is 33 K. This yields a mean overall heat conductance between ADHX and EC of $(UA)_{ADHX,EC}$ of approximately 9 W/K for 'xpr3_L' measurement. By analyzing the other experiments of sorption module 'Size L' this value is found to be the range of 8.5±2W/K as listed in Table S1. As the comparison between 'Size S' and 'Size L' sorption module in Table S1 shows, this value depends on sorption module size.

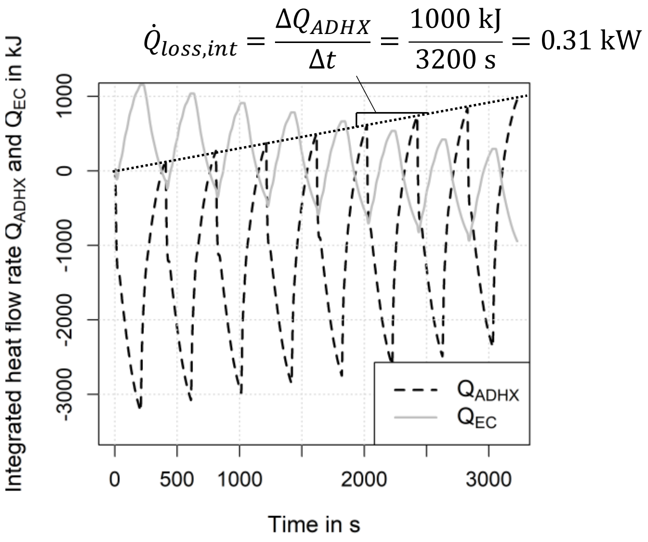

Figure S4. Integrated heat flow rates of ADHX and EC for 'xpr3_L' measurement with calculation of mean internal loss heat flow rate $\dot{Q}_{loss,int}$, related to Results section.

Supplemental Note 2: Temperature Levels and Basic Heat Exchanger Theory

The relevant temperature levels and their relation to the heat and mass transfer resistances of the components of an adsorption module are shown in Figure S5. The original chart as presented by Laurenz[5] was adapted for finite capacity flow rates through the heat exchangers. In case of infinitely high mass flow rates through the heat exchangers (NTU→0, $T_{in}≈T_{out}$) the temperature differences are equal to the ideal case presented by Laurenz[5]. Each heat exchanger is characterized by its temperature levels of heat transfer fluid inlet and outlet, the equilibrium temperature T_{eqi} or the saturation temperature T_{sat}, and the effective heat and mass transfer resistances R. In the real process the temperature differences shown in Figure S5 change continuously within a half cycle since adsorption and desorption are transient processes. All the basic heat exchanger theory that has its origin in the stationary energy balance of an infinitesimal element of a heat exchanger cannot be applied to a process undergoing temperature changes. As detailed in the 'Results' section of the main research article, the adsorption process can be split into a transient switching phase and a quasi-isothermal phase where the adsorptive term in Equation (6) in main research article dominates. Thus, all the temperature levels shown in Figure S5 refer to the mean temperatures in the quasi-isothermal phase.

It must be added that the assumption of a quasi-isothermal phase with negligible temperature changes within a half cycle depends also on the shape of the isotherm. In case of an ideal step-like isotherm this assumption holds true, whereas deviations from the ideal step-like behavior (or a linear isotherm) will challenge this assumption. Thus, it must be checked if Equation (6) in the main research article is fulfilled in case of other isotherm shapes.

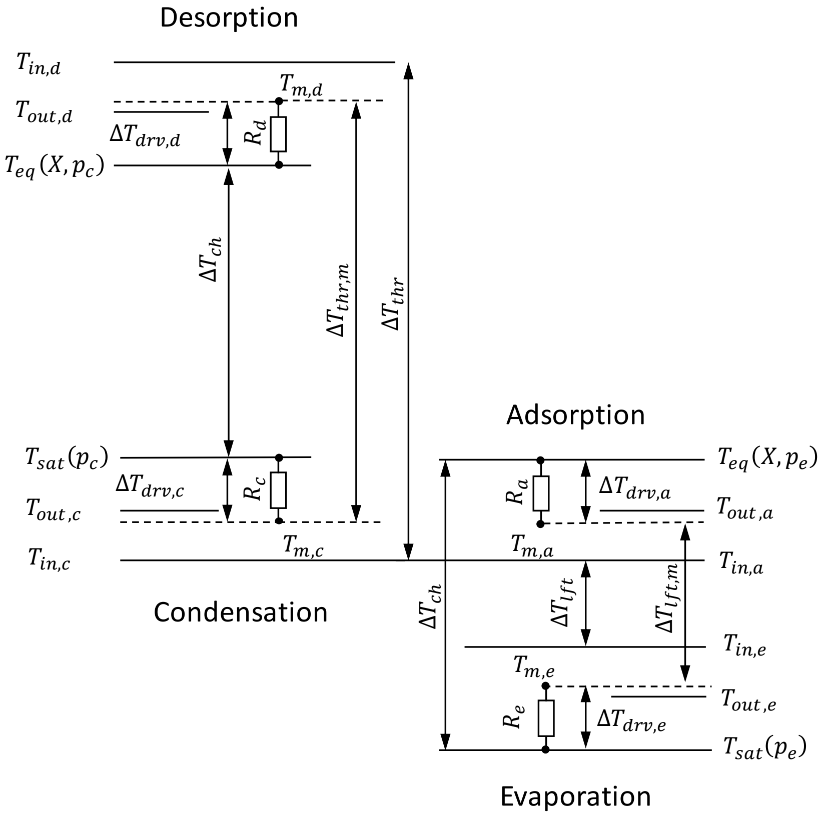

Figure S5. Characteristic temperature difference chart for adsorption heat exchanger and evaporator-condenser, related to Figure 2.

Index 'a' refers to adsorption, 'e' to evaporation, 'd' to desorption and 'c' to condensation.

From a thermodynamic viewpoint, a larger value of ΔT_{ch} allows for a larger temperature lift ΔT_{lft} on the one hand but requires also a larger temperature thrust ΔT_{thr}. As shown in Figure

S5, the temperature lift ΔT_{lft} is defined in Equation (2) in the main article as the temperature difference between the inlet temperatures of the adsorption heat exchanger and the evaporator-condenser secondary (HTF) side in the adsorption half cycle. Additionally, temperature lift and temperature thrust as the temperature differences between the logarithmic mean temperatures of the ADHX and the EC secondary (HTF) side are defined in Equation (S2) and Equation (S3), respectively.

$$\Delta T_{lft,m} = T_{m,a} - T_{m,e} \qquad \text{Equation (S2)}$$

$$\Delta T_{thr,m} = T_{m,d} - T_{m,c} \qquad \text{Equation (S3)}$$

In case of an infinite mass flow rate through the heat exchangers ($\dot{C}_{HTF} \to \infty$), the temperature lift approaches the inlet temperature difference $\Delta T_{lft} \to \Delta T_{lft,in}$. The lower the mass flow rate, the higher the temperature lift $\Delta T_{lft,m}$ will be if the inlet temperatures are set. In contrast, the temperature thrust $\Delta T_{thr,m}$ decreases with a lower mass flow rate.
With this definition of temperature thrust $\Delta T_{thr,m}$ and temperature lift $\Delta T_{lft,m}$, relations between $\Delta T_{thr,m}$ and $\Delta T_{lft,m}$, characteristic temperature difference ΔT_{ch}, and driving temperature differences $\Delta T_{drv,a/d/e/c}$ can be formulated according to Equation (S4) and Equation (S5).

$$\Delta T_{lft,m} = \Delta T_{ch} - \Delta T_{drv,a} - \Delta T_{drv,e} \qquad \text{Equation (S4)}$$

$$\Delta T_{thr,m} = \Delta T_{drv,d} + \Delta T_{drv,c} + \Delta T_{ch} \qquad \text{Equation (S5)}$$

The spatial temperature distribution in the adsorption heat exchanger and the evaporator-condenser during the adsorption half cycle are shown in Figure S6.

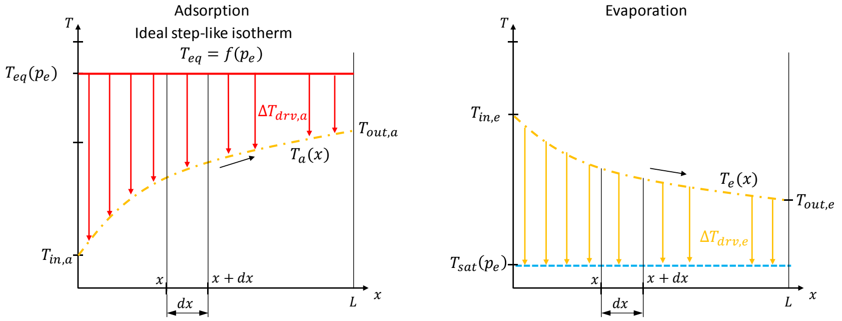

Figure S6. Temperature curves over the heat exchanger length L (x-axis) for adsorption and evaporation, related to Figure 2.
The curves are valid for the quasi-isothermal phase of the process.

The logarithmic mean temperature difference ΔT_{drv} can be calculated as shown in Equation (S6) for evaporation and condensation processes[6]. A similar Equation (8) in the main research article is used for the adsorption and desorption processes with the assumption of $T_{eq}(x) =$ const., as shown in Figure S6, which holds true for working pairs with a step-like isotherm. For such a working pair, $T_{\text{eq}}(X,p) \approx T_{\text{eq}}(p)$ holds true in the relevant loading range.

$$\Delta T_{drv,e/c} = \frac{T_{out,e/c} - T_{in,e/c}}{\ln \dfrac{T_{sat}(p_{e/c}) - T_{in,e/c}}{T_{sat}(p_{e/c}) - T_{out,e/c}}} \qquad \text{Equation (S6)}$$

The equations for heat exchanger effectiveness ϵ, number of transfer units NTU, and the ϵ-NTU relationship for evaporation/condensation and adsorption/desorption in case of an ideal step-like isotherm can be found in Equation (9), Equation (10), and Equation (11) in the main research article.

In case of sorption materials having an ideal linear dependence of characteristic temperature difference and loading, the equilibrium temperatures T''_{eq} and T'_{eq} change over the length of the heat exchanger as shown in Figure S7.
With the additional assumption of a constant heat and mass transfer resistance over the heat exchanger length, the driving temperature difference for adsorption and desorption process with an ideal linear isotherm is given in equation.

$$\Delta T_{drv,a/d} = T_{out,eq} - T_{out,a/d} = T_{in,eq} - T_{in,a/d} \quad \text{Equation (S7)}$$

Since neither $T_{out,eq}$ nor $T_{in,eq}$ are known, the driving temperature difference has to be approximated with $T_{eq}(\bar{X}, p_e)$ and the arithmetic mean of the inlet and outlet temperatures. The mean loading $\bar{X}$ can be calculated with the energy balance of the evaporator-condenser and the equilibrium temperature T_{eq} results then out of Equation (12) in the main research article. Due to the linear shape of the temperature distribution as shown in Figure S7 the driving temperature difference can be calculated with the arithmetic mean of inlet and outlet temperatures with Equation (S8).

$$\Delta T_{drv,a/d} = T_{eq}\left(\bar{X}, p_{e/c}\right) - \frac{T_{in,a/d} + T_{out,a/d}}{2} \quad \text{Equation (S8)}$$

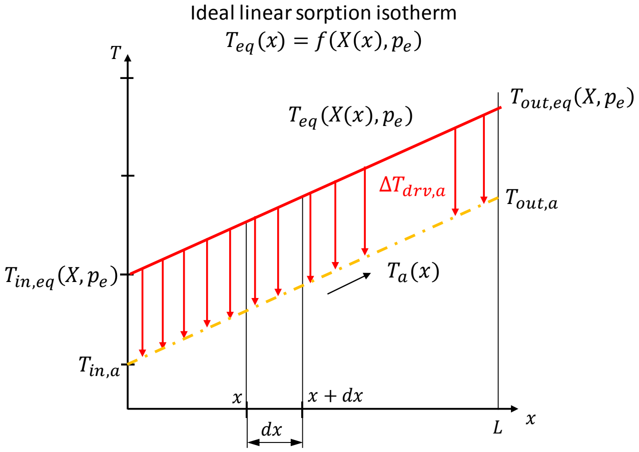

Figure S7. Temperature curves over the heat exchanger length L (x-axis) for ideal linear sorption isotherm, related to Figure 2.

The curves are valid for the quasi-isothermal phase of the process.

The temperature effectiveness ϵ is calculated with equation Equation (S9) according to Shah et al.[6].

$$\epsilon_{a/d} = \frac{T_{out,a/d} - T_{in,a/d}}{T_{in,eq} - T_{in,a/d}} = \frac{T_{out,a/d} - T_{in,a/d}}{\Delta T_{drv,a/d}} \quad \text{Equation (S9)}$$

In case of an ideal linear sorption isotherm the relationship between temperature effectiveness ϵ and number of transfer units NTU is given in Equation (S10) according to Shah et al.[6].

$$\epsilon = \frac{NTU}{1 + NTU} \quad \text{Equation (S10)}$$

Supplemental Note 3: Half Cycle Time and Loading Difference

The total half-cycle time Δt_{hc} is the sum of the contributions from the quasi-isosteric (is) and quasi-isothermal (it) phases as shown in Equation (S11).

$$\Delta t_{\mathrm{hc}} = \Delta t_{\mathrm{is}} + \Delta t_{\mathrm{it}} \qquad \text{Equation (S11)}$$

The contribution from the quasi-isosteric process is calculated with Equation (S12) and Equation (S13) using the ratio of overall thermal capacity and capacity flow rate as a first order approximation. Since the capacity flow rate can be different in adsorption, desorption, evaporation, and condensation four different times for the quasi-isosteric process must be calculated. With conventional device designs adsorption/evaporation and desorption/condensation are bound pairwise, so it is physically impossible to have different times. Here, the slowest process governs Δt_{is}. Hence, the maximum value is taken for $\Delta t_{is,a}$ and $\Delta t_{is,d}$ in Equation (S12) and Equation (S13).

$$\Delta t_{is,a} = \max\left(\frac{C_{p,tot,e}}{\dot{C}_{HTF,e}}, \frac{C_{p,tot,a}}{\dot{C}_{HTF,a}}\right) \qquad \text{Equation (S12)}$$

$$\Delta t_{is,d} = \max\left(\frac{C_{p,tot,c}}{\dot{C}_{HTF,c}}, \frac{C_{p,tot,d}}{\dot{C}_{HTF,d}}\right) \qquad \text{Equation (S13)}$$

In case of an ideal step-like isotherm and constant heat and mass transfer resistances within the components, there is a linear dependence between loading X and time as shown in Figure S8. The loading curves are calculated with Equation (S14) for adsorption and Equation (S15) for desorption, respectively. The timespan Δt_{is} accounts for the quasi-isosteric phase of the process during the rapid temperature change (i. e. heating or cooling the thermal capacity of the components) between adsorption and desorption or evaporation and condensation, respectively. The slope of X(t) in the quasi-isothermal phase $\Delta X/\Delta t_{it}$ is calculated with Equation (7) in the main research article.

$$X_a(t) = X_{min,eqi} + \Delta X_{eqi} \cdot \min\left(\frac{\Delta X}{\Delta t_{it} \cdot \Delta X_{eqi}} \cdot (t - \Delta t_{is}), 1\right) \qquad \text{Equation (S14)}$$

$$X_d(t) = X_{min,eqi} + \Delta X_{eqi} \cdot \left(1 - \min\left(\frac{\Delta X}{\Delta t_{it} \cdot \Delta X_{eqi}} \cdot (t - \Delta t_{is}), 1\right)\right) \qquad \text{Equation (S15)}$$

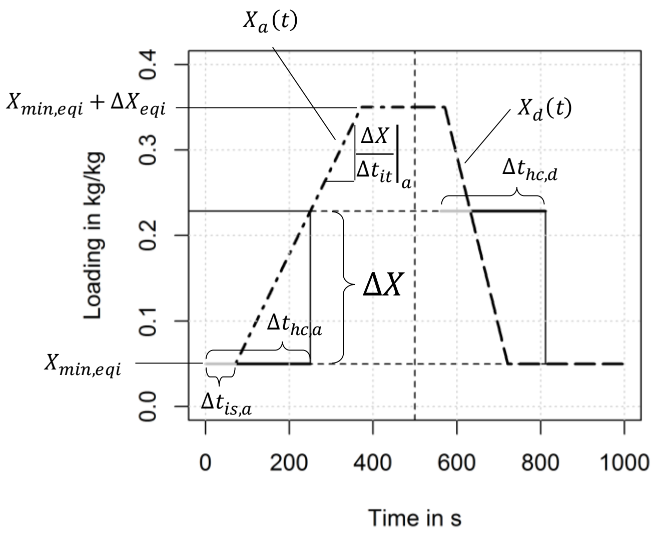

Figure S8. Loading curve in case of ideal step-like sorption isotherm with contributions from the quasi-isosteric (is) and quasi-isothermal (it) phases, related to Results section.

As shown in Figure 2D in the main research article, the SAPO-34-water working pair does not have an ideal step-like isotherm. Thus, the experimental results will deviate from Equation (S14) and Equation (S15) if the relative loading range in the experiment is lower than 20 % or higher than 80 %. Due to the higher driving temperature difference for a relative loading < 20 % the slope $\Delta X/\Delta t_{it}$ will be steeper than predicted with Equation (7) in the main research article. In case of a relative loading > 80 % the driving temperature difference decreases with an increasing loading, thus the slope $\Delta X/\Delta t$ will be flatter than predicted with Equation (7) in the main research article. However, for most practical applications the relative loading will range between 5% and 90%. In this range, the assumption of an ideal step-like isotherm will lead to a correct calculation of the actual loading difference, within an error margin of about 10%. For the sake of simplicity this error is accepted.

In Equation (S16) the total half cycle time Δt_{hc} is calculated with contributions from Equation (S12) and Equation (S14) valid for a linear loading profile.

$$\Delta t_{\mathrm{hc}} = \Delta t_{\mathrm{is,s/e}} + \Delta t_{\mathrm{it,s/e}} = \frac{C_{\mathrm{p,tot,s/e}}}{\dot{C}_{HTF,s/e}} + \frac{M_{sorb} \cdot \Delta h_{ad} \cdot |\Delta X|}{\Delta T_{\mathrm{drv,s/e}}} R_{s/e} \qquad \text{Equation (S16)}$$

An ideal linear sorption isotherm will lead to a non-constant driving temperature difference over the half cycle even during the quasi-isothermal phase. In this case it is assumed that the adsorption process follows the simple exponential function in Equation (S17) and the desorption process follows Equation (S18), as demonstrated by Aristov et al.[7].

$$X_a(t) = X_{min,eqi} + \Delta X_{eqi} \cdot \left(1 - \exp\left(-\frac{t - \Delta t_{is}}{\tau_a}\right)\right) \qquad \text{Equation (S17)}$$

$$X_d(t) = X_{min,eqi} + \Delta X_{eqi} \cdot \exp\left(-\frac{t - \Delta t_{is}}{\tau_d}\right) \qquad \text{Equation (S18)}$$

The time constants $\tau_{a/d}$ will be obtained using Equation (S19). The driving temperature difference $\Delta T_{drv,a/d}$ is calculated in the quasi-isothermal phase of the half cycle with Equation (7)-(10) in the main research article.

$$\tau_{a/d} = \frac{\Delta X_{80-20} \cdot M_{sorb} \cdot \Delta h_{ad}}{R_{a/d}^{-1} \cdot \Delta T_{drv,a/d} \cdot (\ln(1-0.2) - \ln(1-0.8))} = \frac{\Delta X_{80-20} \cdot M_{sorb} \cdot \Delta h_v}{R_{e/c}^{-1} \cdot \Delta T_{drv,e/c} \cdot (\ln(1-0.2) - \ln(1-0.8))} \qquad \text{Equation (S19)}$$

If the half cycle times $\Delta t_{hc,a}$ and $\Delta t_{hc,d}$ are given and the contributions from the quasi-isosteric part $\Delta t_{is,a}$ and $\Delta t_{is,d}$ are calculated, the loading difference ΔX can be calculated iteratively as illustrated in Figure S9.

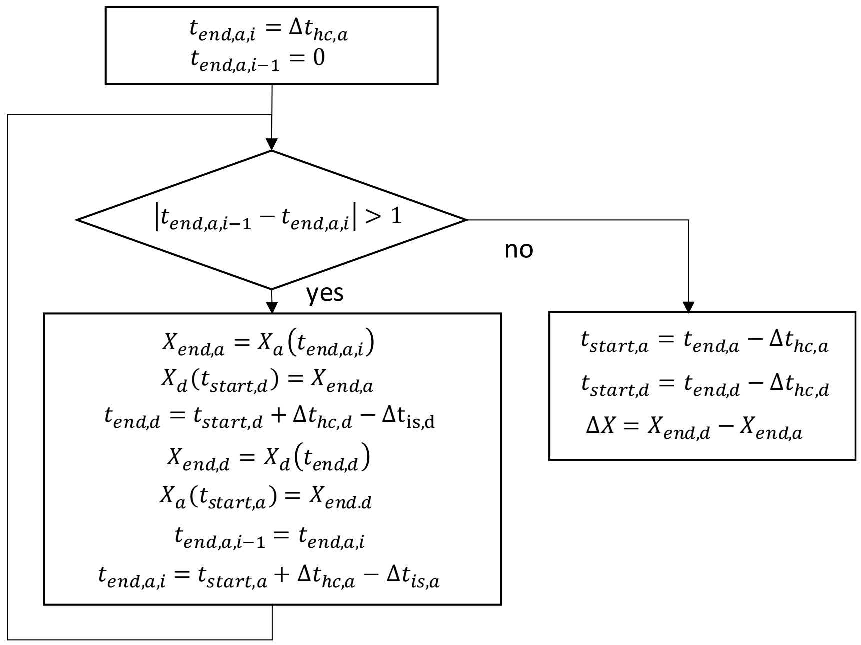

Figure S9. Flow chart of iterative procedure to calculate the loading difference ΔX with given half cycle times $\Delta t_{hc,a}$ and $\Delta t_{hc,d}$, related to Results section.

Supplemental Note 4: Effective Heat and Mass Transfer Resistances

Measurement data from sorption module measurements can be used to calculate effective heat and mass transfer resistances as illustrated in Figure 2B/C in the main research article. A previously published calculation procedure[4] requires an equilibrium model to calculate the equilibrium temperature $T_{eq}(X,p)$. Besides this calculation procedure the characteristic temperature difference ΔT_{ch} in Equation (12) in the main research article can be exploited, which makes a more complex equilibrium model obsolete and makes the calculation easier. For this purpose, Equation (10) in the main research article can be transformed to Equation (S20) yielding the effective heat and mass transfer resistances of adsorption (a), desorption (d), evaporation (e), and condensation (c). The capacity flow rate $\dot{C}_{HTF}$ is the product of mass flow rate and specific heat capacity of the heat transfer fluid (water).

$$R_{a/d/c/e} = \frac{1}{NTU_{a/d/c/e} \cdot \dot{C}_{HTF,a/d/c/e}} \qquad \text{Equation (S20)}$$

The number of transfer units NTU in Equation (S20) can be calculated with Equation (11) in the main research article. The temperature effectiveness ϵ is calculated with Equation (9) in the main research article in case of a step like isotherm. In case of a linear isotherm, Equation (S9) and Equation (S10) are used for the adsorption heat exchanger ($NTU_{a/d}$ and $\epsilon_{a/d}$).
In case of SAPO-34, the equilibrium temperature $T_{eq}(X,p)$ in Equation (8) in the main research article can be calculated with Equation (12) in the main research article using a constant mean value of 34 K for the characteristic temperature difference ΔT_{ch}.
The overall effective heat and mass transfer resistance of the adsorption module is calculated with Equation (S21) as the sum of the effective heat and mass transfer resistances of adsorption heat exchanger and evaporator-condenser. This results in a value $R_{mod,a/e}$ in the adsorption/evaporation half cycle and $R_{mod,d/c}$ in the desorption/condensation half cycle.

$$R_{mod,a/e,d/c} = R_{a/d} + \frac{\Delta h_{ad}}{\Delta h_v} \cdot R_{e/c} \qquad \text{Equation (S21)}$$

Prior to manufacturing adsorption heat exchangers, small-scale samples as shown in Figure S1 were manufactured to study the adsorption dynamics as presented previously by Velte et al.[2]. The samples were measured in an adsorption dynamics measurement set-up as described by Velte et al.[2] with the large pressure jump (LPJ) method. Prior to the LPJ measurement the small-scale sample under investigation is attached to the temperature controlled carrier plate of the set-up. First, the carrier plate is heated to 95 °C and the sample desorbs at a water vapor pressure of 42.5 mbar. Then the valves connecting the measurement chamber with the dosing chamber are closed and the sample is cooled down to $T_{car} = 40\ °C$. By cooling down the sample the remaining water vapor in the chamber is adsorbed until an equilibrium state is reached. Depending on the adsorbent mass the pressure in the measurement chamber drops to values below 5 mbar during the cool-down. At the same time, the dosing chamber is filled with water vapor at a pressure of 23.4 mbar. After the sample reached its equilibrium state the valve connecting the measurement chamber and the dosing chamber is opened and the sample adsorbs the water vapor. Since measurement chamber and dosing chamber are a closed volume during the measurement, this adsorption process leads to a pressure drop in this closed volume. If the adsorbent mass of the sample is sufficiently small, the pressure drop is < 4 mbar. Then, the measurement can be seen as a quasi-isobaric adsorption process (4→1 in Figure 1B in the main research article) The amount of adsorbed water M_{wf} can be calculated with the ideal gas law using the measured pressure p_{vap}, the known volume of the set-up, and the measured vapor temperature. The heat flow rate of the LPJ experiments is calculated with Equation (S22) and Equation (S23), with $\dot{Q}_s$ being the sorptive heat flow rate and $\dot{Q}_{C_p}$ being the capacitive heat flow rate[8]. For the calculation of the capacitive heat flow rate the surface temperature T_{srf} is taken as the mean temperature of the sample as a first order approximation[8]. This temperature is measured with an infrared sensor.

$$\dot{Q}_s = M_{sorb} \cdot \frac{dX}{dt} \cdot \Delta h_{ad} = \frac{dM_{wf}}{dt} \cdot \Delta h_{ad} \approx \underbrace{\frac{M_{wf}(t_{80}) - M_{wf}(t_{20})}{t_{80} - t_{20}} \cdot \Delta h_{ad}}_{\text{valid between 20\% and 80\% relative loading}} \qquad \text{Equation (S22)}$$

$$\dot{Q}_{C_p} = \left(C_{p,tot} + X \cdot M_{sorb} \cdot c_{p,adb}\right) \cdot \frac{dT_{srf}}{dt} \approx \underbrace{C_{p,tot} \cdot \frac{T_{srf}(t_{80}) - T_{srf}(t_{20})}{t_{80} - t_{20}}}_{\text{valid between 20\% and 80\% relative loading}} \quad \text{Equation (S23)}$$

The effective heat and mass transfer resistance of small-scale samples is calculated with Equation (S24). The temperature difference between equilibrium temperature T_{eq} and carrier plate temperature T_{car} can be expressed in terms of characteristic temperature difference with $\Delta T_{lft} = T_{car} - T_{sat}(p_{vap})$ and the constant mean value $\Delta \overline{T}_{ch} = 34\ \mathrm{K}$ in case of SAPO-34.

$$R_{a,Ssc} = \frac{T_{eq} - T_{car}}{\dot{Q}_s + \dot{Q}_{C_p}} = \frac{\Delta T_{ch} - \Delta T_{lft}}{\dot{Q}_s + \dot{Q}_{C_p}} \quad \text{Equation (S24)}$$

The area-scaled, effective heat and mass transfer resistance of the two small-scale samples is shown in Figure S10.

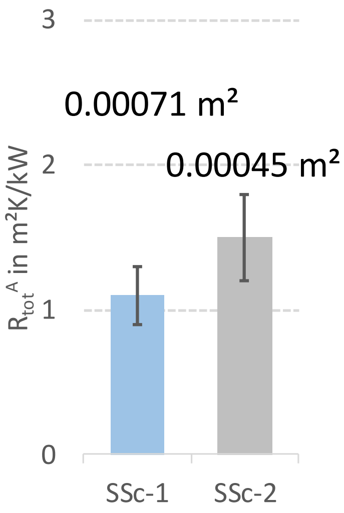

Figure S10. Effective heat and mass transfer resistance of small-scale samples scaled with the sample area, related to Discussion section.

The reference area of each sample is shown above the error bars. Error bars are calculated with Gaussian error propagation method.

Since the measurement of the small-scale samples is similar to a quasi-isobaric adsorption process, the area-scaled effective heat and mass transfer resistance of the small-scale samples can be compared with the values obtained from sorption module measurements as shown in Figure 3E/F in the main research article.

Supplemental Note 5: Sizing Problem

The relevant parameters and variables of the sizing problem are shown in Figure S11.

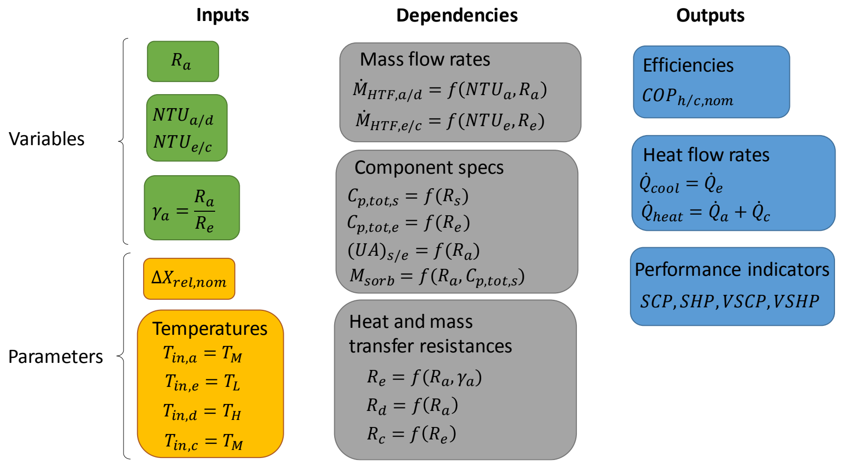

Figure S11. Overview of inputs, dependencies, and outputs relevant for the sizing process of an adsorption module related to Figure 4.

The inputs are clustered into variables that are varied in the sizing process and parameters that are fixed. The overall heat and mass transfer resistance of the adsorption heat exchanger during the adsorption half cycle R_a is chosen as the variable in the sizing process. It would also be possible to choose any of R_e, R_c, R_d yielding the same results. The NTU of the adsorption heat exchanger and the evaporator condenser is varied within a range of 0.2…3. The lower limit of 0.2 is set according to the expected pressure losses of the components. Since this lower limit depends on the geometry of the hydraulic part of the adsorption heat exchanger this value must be critically reviewed for each type of adsorption heat exchanger. The ratio of the heat and mass transfer resistances of the adsorption heat exchanger and the evaporator-condenser γ_a is varied within a range of 0.1…4, restricting the possible combinations of component sizes.

Since the efficiency strongly depends on the loading spread ΔX, and thus, on the half cycle time, we must define a relative loading range $\Delta X_{rel,nom}$ to compare the results of the sizing process on the same basis. In general, any value between 1 % and 99 % might be chosen, the practically relevant values range around 50% and 90 % of the equilibrium loading spread. For our example here a value of 80 % is chosen for the nominal operating point of the sizing process. This results in a nominal half cycle time that depends on the component size and mass flow rates. Once the sizing process is completed and the adsorption module operates in a real-life process, the half cycle time and the mass flow rates are the most important control parameters, and they can be adjusted to meet heat flow requirements that deviate from the nominal operating point. The component inlet temperatures $T_{in,a}$, $T_{in,d}$, $T_{in,e}$, and $T_{in,c}$ must be chosen according to the requirements of the application in terms of T_L, T_M, and T_H. Furthermore, the condition $\Delta T_{lft,in}$ < $\Delta T_{thr,in}$ must be met.

In the next step, the dependent variables and parameters are calculated. The mass flow rates are calculated from the NTU and the overall heat and mass transfer resistance of the component with Equation (10) in the main research article. In general, the main component specifications in terms of effective thermal masses $C_{p,tot,s}$ and $C_{p,tot,e}$, internal loss between adsorption heat exchanger and evaporator-condenser $UA_{ADHX,EC}$, and adsorbent mass M_{sorb} are somehow related to the heat and mass transfer resistances. If the component specifications are known for a specific design as it is the case here, a simple linear dependency can be assumed as shown in Figure S12 and Figure S13. If the design is new or heat and mass transfer resistances are not available, these dependencies might be calculated or estimated as suggested by Laurenz[5].

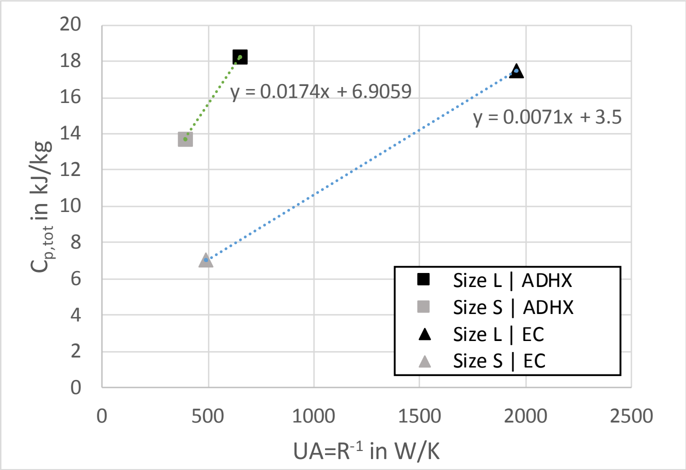

Figure S12. Overall thermal capacity depending on overall heat and mass transfer resistance of 'Size S' components and 'Size L' module components related to Results section.

The formulas show the coefficients of the linear interpolation.

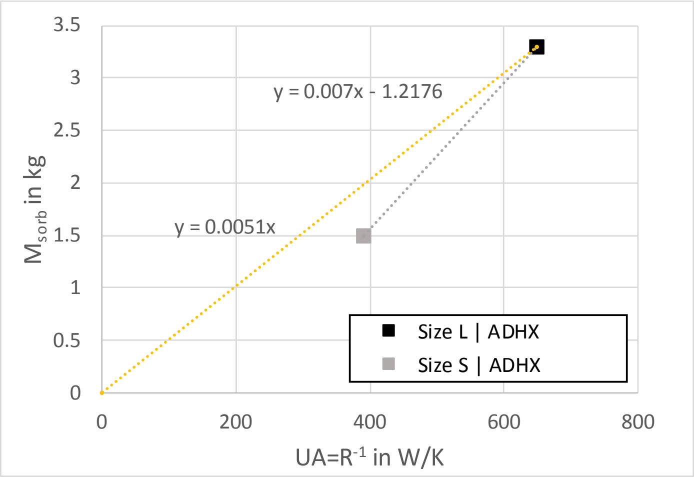

Figure S13. Adsorbent mass depending on overall heat and mass transfer resistance of 'Size S' ADHX and 'Size L' ADHX related to Results section.

The formulas show the coefficients of the linear interpolation.

Supplemental Note 6: Quantitative Comparison with Detailed Numerical Model Prediction Quality

The prediction quality of the calculation method is compared to a detailed transient numerical model as presented by Velte[9] earlier. Velte modelled the 'Size L' adsorption module with ADHX and EC having a one-dimensional spatial discretization of the heat transfer fluid in fluid flow direction. Metal parts of the ADHX and its fibrous structure directly crystallized with SAPO-34 are also modelled with one-dimensional spatial discretization in fluid flow direction. All nodes of the three domains are connected with heat transfer resistances accounting for conduction and convection. The limiting mass transfer mechanism was identified to be adsorbate diffusion within the compact layer of SAPO-34. As detailed by Füldner[10] and Velte et al.[2] the diffusion equations for the mass transfer in the adsorbed phase can be simplified to the linear driving force approach[2], which is used by Velte[9] to model the mass transfer in the 'Size L' sorption module.

The experimental results of 'Size L' sorption module and the results of the detailed transient numerical model along with the results of the calculation method presented here are shown in Figure S14. It is obvious that the detailed transient numerical model is able to predict all experiments within the measurement uncertainty, whereas one outlier (xpr3_L) is observed if the experiment is predicted with the calculation method presented here. The quantitative comparison in Table S4 yields a 4 times higher prediction quality regarding the efficiency and a 7 times higher prediction quality regarding the heat flow rate of the detailed transient numerical model compared to the calculation method presented here. However, the decisive difference between the results of the detailed transient numerical model to the calculation method presented here is the adaption of the adsorbate diffusion coefficient D_{adb} to match the experimental results. As listed in Table S4 values from $9 \cdot 10^{-12}$ to $14 \cdot 10^{-12}$ m^2/s had to be chosen to achieve the very good match to the experimental data. If the effective heat and mass transfer resistances in adsorption R_a and desorption R_d used in the calculation method presented here are reduced by 30 % in case of xpr3_L, a comparable prediction quality is achieved as shown in Figure S14 ('calc*').

This demonstrates that an even higher prediction quality can be achieved with the presented method, when sufficient data is available to define transfer resistances that vary with loading, pressure or temperature. Since the aim of this publication is rather to show the method in principle, details on how to find well-suited variable effective resistances are not discussed in this work.

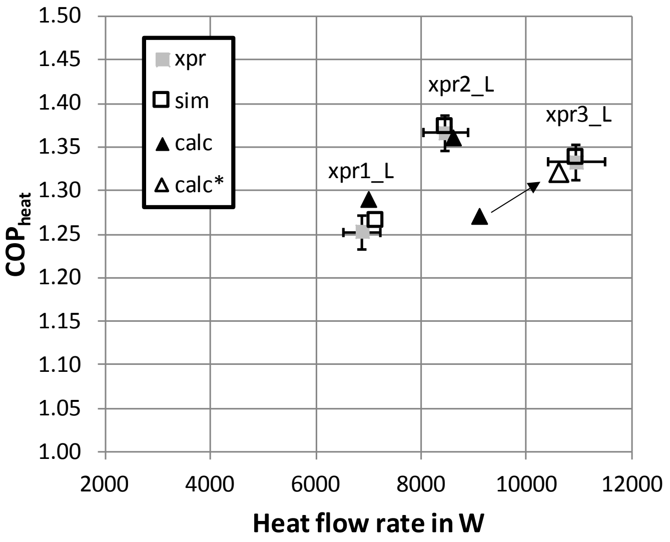

Figure S14. Efficiency and heat flow rate of 'Size L' sorption module obtained from experiments (xpr), detailed transient numerical simulation (sim), and calculation method presented here (calc) related to Figure 3.

The calculation result with adapted effective heat and mass transfer resistances (calc*) in case of xpr3_L is shown additionally. Error bars of the experimental data points are calculated with Gaussian error propagation method.

Table S4. Quantification of prediction quality of detailed transient numerical model and calculation method presented here related to Figure 3.

The bracketed values for xpr3_L refer to the calculation with adapted $\boldsymbol{R_a}$ and $\boldsymbol{R_d}$.

	COP	COP	COP	COP	COP	$\dot{Q}_{heat}$	$\dot{Q}_{heat}$	$\dot{Q}_{heat}$	$\dot{Q}_{heat}$	$\dot{Q}_{heat}$	D_{adb}
	xpr	sim	$(y-y')^2$	calc	$(y-y')^2$	xpr	sim	$(y-y')^2$	calc	$(y-y')^2$	in 10^{-12} m^2/s
**xpr1_L**	1.25	1.27	0.0002	1.29	0.0014	6.9	7.1	0.0625	7	0.0141	9
**xpr2_L**	1.37	1.37	0.0001	1.36	0.0000	8.5	8.4	0.0001	8.6	0.0196	11
**xpr3_L (xpr3_L)***	1.33	1.34	0.0000	1.27 (1.34)*	0.0039	10.9	10.9	0.0003	9.1 (10.6)*	3.4151	14
**RMSD**			**0.01**		**0.04**			**0.14**		**1.07**	
**CV**			**0.7 %**		**3.2 % (1.8 %)***			**1.7 %**		**12.7 % (2.5 %)***	

* results for calculation with adapted R_a and R_d

SUPPLEMENTAL REFERENCES

1. Bauer, J., Herrmann, R., Mittelbach, W., and Schwieger, W. (2009). Zeolite/aluminum composite adsorbents for application in adsorption refrigeration. International Journal of Energy Research *33,* 1233–1249. 10.1002/er.1611.
2. Velte, A. Füldner, G. Laurenz, E., Schnabel, L. (2017). Advanced Measurement and Simulation Procedure for the Identification of Heat and Mass Transfer Parameters in Dynamic Adsorption Experiments. Energies *10,* 1130. 10.3390/en10081130.
3. Wittstadt, U., Füldner, G., Laurenz, E., Warlo, A., Große, A., Herrmann, R., Schnabel, L., and Mittelbach, W. (2017). A novel adsorption module with fiber heat exchangers: Performance analysis based on driving temperature differences. Renewable Energy *110,* 154–161. 10.1016/j.renene.2016.08.061.
4. Velte, A., Joos, L., and Füldner, G. (2022). Experimental Performance Analysis of Adsorption Modules with Sintered Aluminium Fiber Heat Exchangers and SAPO-34-Water Working Pair for Gas-Driven Heat Pumps: Influence of Evaporator Size, Temperatures, and Half Cycle Times. Energies *15,* 2823. 10.3390/en15082823.
5. Laurenz, E. (2021). Frequency response analysis of heat and mass transfer in adsorbent composites and simplified performance estimation for heat transformation applications, TUHH Universitätsbibliothek. 10.15480/882.3836.
6. Shah, R.K., and Sekulić, D.P. (2003). Fundamentals of heat exchanger design (Wiley-Interscience).
7. Aristov, Y.I., Glaznev, I.S., and Girnik, I.S. (2012). Optimization of adsorption dynamics in adsorptive chillers: Loose grains configuration. Energy *46,* 484–492. 10.1016/j.energy.2012.08.001.
8. Velte, A., Laurenz, E., Rustam, L., P.C. Hügenell, P., Henninger, M., Seiler, J., and Füldner, G. (2023). Adsorption dynamics and hydrothermal stability of MOFs aluminium fumarate, MIL-160 (Al), and CAU-10-H, and zeotype TiAPSO for heat transformation applications. Appl. Therm. Eng. *227,* 120336. 10.1016/j.applthermaleng.2023.120336.
9. Velte, A. (2019). Experimentelle Arbeiten und Entwicklung von numerischen Modellen zur Analyse und Optimierung von erweiterten Adsorptionskreisläufen für die Wärmeversorgung von Gebäuden. Dissertation, Universität Freiburg. 10.6094/UNIFR/154691.
10. Füldner, G. (2015). Stofftransport und Adsorptionskinetik in porösen Adsorbenskompositen für Wärmetransformationsanwendungen. Dissertation, Universität Freiburg.
